# Supplementary material for: Discovery of Dolutegravir Derivative against Liver Cancer via Inducing Autophagy and DNA Damage
Source: Molecules. 2024 Apr 13;29(8):1779. doi: 10.3390/molecules29081779 (PMC11052077; doi:10.3390/molecules29081779)

Discovery of dolutegravir derivative against liver cancer via inducing autophagy and DNA damage

Xi-xi Hou[†a], Dong Yan[†b], Ziyuan Wu[†b], Long-fei Mao[b], Huili Wang[c], Yajie Guo[d*], Jianxue Yang [a*]

[a]The First Affiliated Hospital, and College of Clinical Medicine of Henan University of Science and Technology, Luoyang, 471003, China

[b]College of Basic Medicine and Forensic Medicine, Henan University of Science and Technology, 263 Kaiyuan Road, Luoyang 471003, China

[c]University of North Carolina Hospitals, 101 Manning Dr, Chapel Hill, Orange County, NC 27599, USA

[d]Department of Emergency, The Eighth Affiliated Hospital, Sun Yat-Sen University, Shenzhen, 518033, China

†These authors have contributed equally to this work as first co-authors

*Corresponding authors:

Yajie Guo, Department of Emergency, The Eighth Affiliated Hospital, Sun Yat-Sen University, Shenzhen, 518033, China. E-mail: guoyj36@mail.sysu.edu.cn

Jian-xue Yang, The First Affiliated Hospital, and College of Clinical Medicine of Henan University of Science and Technology, Luoyang, 471003, China. E-mail: Docyix1969@126.com

Synthesis of (4R,12aS)-3,4,6,8,12,12a-hexahydro-7-methoxy-4-methyl-6,8-dioxo-2H-pyridine [1',2': 4,5]pyrazino[2,1-b][1,3]oxazine-9-carboxylic acid (Compound 3)

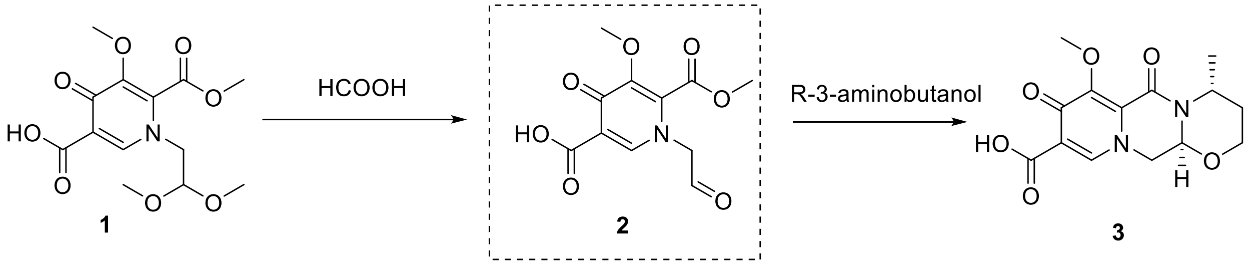

In a reaction flask, 1-(2,2-dimethoxyethyl)-1,4-dihydro-3-methoxy-4-oxo-2,5-pyridinedicarboxylic acid-2-methyl ester (compound **1**, 6.2 g) was added to 100 mL of anhydrous formic acid. The reaction proceeded at 65 ° C with stirring and under an argon atmosphere. The reaction was completed at about 3 hrs when the starting material was used up as monitored with TLC. Under the vacuum, concentrated and evaporate formic acid at 45~55 ° C to give a crude oil (Compound **2**).

Add 100 mL of acetonitrile to the crude compound **2** with stirring. R-3-aminobutanol (2.5 g) was introduced and stirred for 10 min. The temperature was then raised to an internal temperature of 80-82 ° C and stirring continued for 2.5 h. The reaction completed at this time as monitored with TLC. Concentrate to remove most of the solvent at 45 °C. Added 100 mL of dichloromethane, and then 50mL water while stirring. The pH was adjusted to 1-2 using 2N HCl and stirred for an additional 10 min. The lower organic phase was separated, and the organic portion was washed three times with 20 mL of saturated NaCl solution. Concentrated the mixture under vacuum to give a crude product. It was then purified by recrystallization using methanol to gave 4.11 g of pure product (Compound **3**); ^{1}H NMR (400 MHz, $CDCl_3$): δ 8.43 (s, 1H), 5.30 (t, J = 4.0 Hz,1H), 5.02 (t, J = 4.0Hz, 1H), 4.41 (dd, J_1 = 4.0Hz, J_2 = 4.0Hz, 1H), 4.27 (dd, J_1 = 8.0Hz, J_2 = 4.0Hz, 1H), 4.08 (s, 3H), 4.03-3.99 (m, 2H), 2.25-2.16 (m, 1H), 1.56 (d, J = 12.0Hz, 1H), 1.39 (d, J = 8.0Hz, 3H). ^{13}C NMR (100 MHz, $CDCl_3$): δ 176.39, 165.85, 155.00, 153.90, 142.78,

130.66, 116.08, 75.97, 62.65, 61.48, 53.89, 44.93, 29.37, 16.06

General synthetic procedure for compound 4

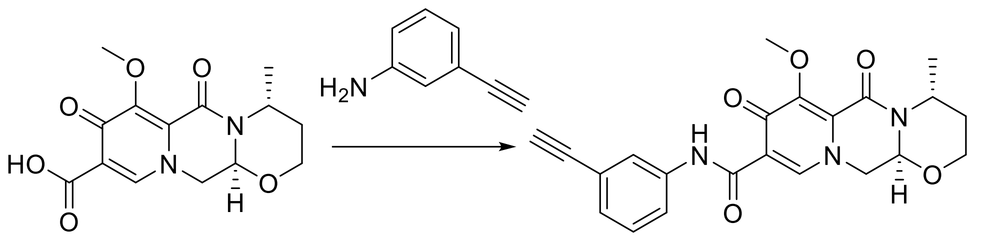

3　　　　　　　　　　　　　　　　4

Compound **3** (10 g), 3-aminophenylacetylene (7.4 g), HATU (26 g), DIPEA (16.3 g) and solvent DMF 400ml were added to a 1000mL reaction flask at room temperature and stirred under nitrogen protection. Thin layer chromatography (TLC) was used for monitoring. After 28 hours, the reaction completed. DMF was removed by vacuum concentration, dichloromethane (200mL) and water (100mL) were added to the reaction solution. Separate the organic phase after stirring. The organic solutions was washed with saturated sodium chloride (100mLx2) , and the viscous brownish yellow liquid was obtained by vacuum distillation. Under ultrasonic vibration, methanol was slowly added drop by drop, and solid precipitated. After that, it was left to stand, filtered and dried to obtain the compound **4**, 7.92g. ^{1}H NMR (400 MHz, DMSO-d_6) δ 12.55 (s, 1H), 8.70 (s, 1H), 7.96 (s, 1H), 7.60 (d, J = 8.0 Hz, 1H), 7.38 (t, J = 8.0 Hz, 1H), 7.22 (d, J = 8.0 Hz, 1H), 5.39 (dd, J_1 = 4.0 Hz, J_2 = 4.0 Hz, 1H), 4.80-4.70 (m, 1H), 4.62 (dd, J_1 = 4.0 Hz, J_2 = 4.0 Hz, 1H), 4.40 (dd, J_1 = 8.0 Hz, J_2 = 4.0 Hz, 1H), 4.21 (s, 1H), 3.98 (d, J = 4.0Hz, 1H), 3.90-3.86 (m, 1H), 3.84 (s, 3H), 2.09-1.93 (m, 1H), 1.53 (d, J =12.0 Hz, 1H), 1.29 (d, J = 8.0 Hz, 3H). ^{13}C NMR (100MHz, DMSO-d_6): 173.7, 161.8, 155.1, 152.7, 143.1, 138.5, 132.2, 130.3, 129.5, 127.0, 122.4, 122.3, 120.3, 118.5, 117.4, 83.2, 80.7, 75.7, 61.7, 60.2, 52.3, 29.1, 15.6.

General synthetic procedure for compounds 5a-5z and 6a-6h

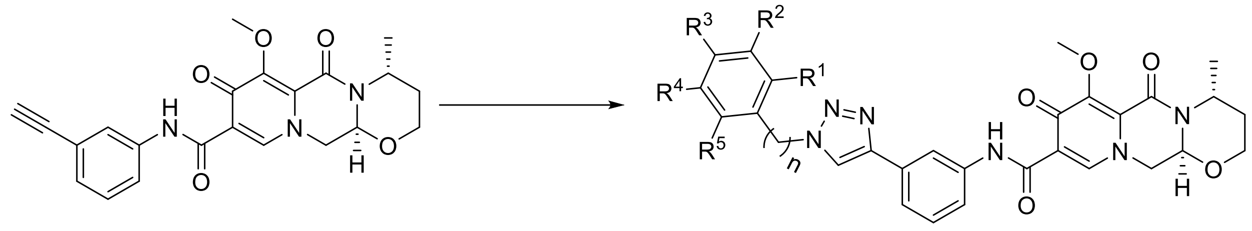

In the reaction flask, compound **4** (3 mmol), substituted azide (3.6 mmol), TERT butanol 100 mL, water 100 mL, tetrahydrofuran 100 mL, anhydrous copper sulfate (6 mmol) and sodium ascorbate (1 mmol) were successively added. The mixture was stirred and refluxed at 70°C for 6 hours. After completion of the reaction (monitored by TLC), dichloromethane (100 mL×3) was used for extraction. The organic solutions were combined and washed with saturated sodium chloride aqueous solution (100 mL×2). The combined organic layer was washed with brine (100 mL×2), dried over sodium sulfate, and concentrated in vacuo to give the crude product. Recrystallization in ethyl acetate produced the desired compound which was pure enough for further characterization and anti-tumor study.

Compound 5a: Pure 98.9%. white solid, – MS (ESI): *m/z* (%) = 545 $[M + H]^+$; ^1H NMR (400 MHz, DMSO-d_6): 12.62 (s, 1 H), 9.17 (s, 1 H), 8.73 (s, 1 H), 8.24 (s, 1 H), 7.90 (d, *J* = 24.0 Hz, 2 H), 7.68 (d, *J* = 16.0 Hz, 3 H), 7.50 (s, 2 H), 5.40 (s, 1 H), 4.80 (s, 1 H), 4.65 (s, 1 H), 4.43 (s, 1 H), 3.98 (s, 1 H), 3.89-3.86 (m, 4 H), 1.98 (s, 1 H), 1.54 (s, 1 H), 1.30 (d, *J* = 8.0 Hz, 3 H). ^{13}C NMR (100 MHz, DMSO-d_6): 174.2, 162.2, 155.6, 153.4, 153.2, 147.1, 143.6, 139.5, 131.3, 130.8, 130.2, 126.4, 126.0, 123.5, 121.4, 120.0, 118.1, 117.7, 117.6, 116.9, 76.3, 62.2, 60.7, 55.3, 52.8, 44.8, 29.6, 16.1.

Compound 5b: Pure 97.2%. white solid, – MS (ESI): *m/z* (%) = 602 $[M + H]^+$; ^1H NMR (400 MHz, DMSO-d_6): 12.61 (s, 1 H), 9.16 (s, 1 H), 8.73 (s, 1 H), 8.24 (s, 1 H), 8.12 (s, 1 H), 8.08 (d, *J* = 4.0 Hz, 2 H), 7.84 (d, *J* = 8.0 Hz, 1 H), 7.72 (d, *J* = 8.0 Hz, 1 H), 7.50 (t, *J* = 4.0 Hz, 1 H), 5.40 (s, 1 H), 4.79 (t, *J* = 8.0 Hz,1 H), 4.64 (d, *J* = 12.0 Hz, 1 H), 4.43 (t, *J* = 4.0 Hz, 1 H), 4.08 (s, 3 H), 3.99 (t, *J* = 12.0 Hz, 1 H), 3.86 (s, 3 H), 2.01-1.95 (m, 1 H), 1.53 (d, *J* = 16.0 Hz, 1 H), 1.30 (d, *J* = 4.0 Hz, 3 H); ^{13}C NMR (100 MHz, DMSO-d_6): 155.1, 152.7, 151.5, 148.2, 146.3, 143.0, 138.9, 130.7, 130.4, 130.3, 129.7, 125.9, 123.4, 121.0, 119.5, 117.6, 116.4, 116.3, 108.3, 75.8, 61.7, 60.2, 57.1, 52.3, 44.3, 29.1, 15.6。

Compound 5c: Pure 98.1%. white solid, – MS (ESI): *m/z* (%) = 595 $[M + H]^+$; ^1H NMR (400 MHz, DMSO-d_6): 12.62 (s, 1 H), 9.09 (s, 1 H), 8.72 (s, 1 H), 8.23 (s, 1 H), 8.08 (d, *J* = 8.0 Hz, 1 H), 7.98 (t, *J* = 8.0 Hz, 1 H), 7.90 (t, *J* = 8.0 Hz, 1 H), 7.84 (t, *J* = 8.0 Hz, 2 H), 7.68 (d, *J* = 8.0 Hz, 1 H), 7.49 (t, *J* = 8.0 Hz, 1 H), 5.39 (s, 1 H), 4.79 (t, *J* = 4.0 Hz, 1 H), 4.62 (d, *J* = 12.0 Hz, 1 H), 4.42 (dd, J_1 = 8.0 Hz, J_2 = 4.0 Hz, 1 H), 3.98 (t, *J* = 12.0 Hz, 1 H), 3.89 (s, 1 H), 3.85 (s, 3 H), 2.01-1.94 (m, 1 H), 1.52 (d, *J* = 12.0 Hz, 1 H), 1.29 (d, *J* = 8.0 Hz, 3 H). ^{13}C NMR (100 MHz, DMSO-d_6): 173.7, 161.7, 155.1, 152.7, 146.2, 143.0, 139.0, 134.2, 134.0, 131.2, 130.8, 130.3, 129.8, 129.2, 127.5, 124.3, 120.8, 119.5, 117.6, 116.4, 75.8, 61.7, 60.2, 52.3, 44.3, 29.1, 15.6。

Compound 5d: Pure 96.7%. white solid, – MS (ESI): *m/z* (%) = 595 $[M + H]^+$; ^1H NMR (400 MHz, DMSO-d_6): 12.65 (s, 1 H), 9.55 (s, 1 H), 8.72 (s, 1 H), 8.25 (d, *J* = 8.0 Hz, 2 H), 8.19 (s, 1 H), 8.04 (d, *J* = 8.0 Hz, 2 H), 7.88 (d, *J* = 8.0 Hz, 1 H), 7.71 (d, *J* = 8.0 Hz, 1 H), 7.51 (t, *J* = 8.0 Hz, 1 H), 5.40 (s, 1 H), 4.79 (t, *J* = 4.0 Hz,1 H), 4.64 (d, *J* = 12.0 Hz, 1H), 4.42 (d, *J* = 8.0 Hz, 1 H), 3.98 (t, *J* = 12.0 Hz, 1 H), 3.89 (s, 1 H), 3.86 (s, 3 H), 2.01-1.94 (m, 1 H), 1.53 (d, *J* = 12.0 Hz, 1 H), 1.29 (d, *J* = 8.0 Hz, 3 H); ^{13}C NMR (150 MHz, DMSO-d_6):174.3, 162.2, 155.6, 153.2, 147.8, 143.5, 139.8, 139.5, 131.2, 130.4, 129.3, 127.7, 120.8, 120.5, 120.1, 118.1, 116.9, 76.3, 62.2, 60.7, 52.9, 44.8, 29.6, 16.1。

Compound 5e: Pure 98.2%. yellow solid, – MS (ESI): *m/z* (%) = 586 $[M + H]^+$; ^1H NMR (400 MHz, DMSO-d_6): 12.63 (s, 1 H), 9.12 (s, 1 H), 8.71 (s, 1 H), 8.20 (t, *J* = 8.0 Hz, 2 H), 7.95 (d, *J* = 8.0 Hz, 1 H), 7.84 (d, *J* = 8.0 Hz, 1 H), 7.73 (dd, J_1 = 8.0 Hz, J_2 = 4.0 Hz, 2 H), 7.50 (t, *J* = 8.0 Hz, 1 H), 5.39 (s, 1 H), 4.79 (s, 1 H), 4.62 (d, *J* = 12.0 Hz, 1 H), 4.41 (d, *J* = 8.0 Hz, 1 H), 3.98 (t, *J* = 12.0 Hz, 1 H), 3.89 (s, 1 H), 3.85 (s, 3 H), 2.26 (s, 3 H), 1.97 (s, 1 H), 1.53 (d, *J* = 12.0 Hz, 1 H), 1.29 (d, *J* = 8.0 Hz, 3 H); ^{13}C NMR (100 MHz, DMSO-d_6): 173.7, 161.7, 155.1, 152.7, 150.7, 146.4, 143.0, 139.0, 137.6, 130.9, 130.8, 130.3, 129.8, 128.1, 128.0, 125.6, 123.9, 120.8, 119.5, 117.6, 116.4, 75.8, 61.7, 60.2, 52.3, 44.3, 29.1, 15.6, 13.9。

Compound 5f: Pure 95.4%. white solid, – MS (ESI): *m/z* (%) = 541 $[M + H]^+$; ^1H NMR (400 MHz, DMSO-d_6): 12.63 (s, 1 H), 9.36 (s, 1 H), 8.72 (s, 1 H), 8.18 (s, 1 H), 7.88 (d, *J* = 8.0 Hz, 1 H), 7.84 (s, 1 H), 7.78 (d, *J* = 8.0 Hz, 1 H), 7.71 (d, *J* = 8.0 Hz, 1 H), 7.50 (dd, J_1 = 8.0 Hz, J_2 = 8.0 Hz, 2 H), 7.34 (d, *J* = 4.0 Hz, 1 H), 5.41-5.39 (m, 1 H), 4.83-4.77 (m, 1 H), 4.66-4.62 (m, 1 H), 4.45-4.40 (m, 1 H), 3.99 (t, *J* = 12.0 Hz, 1 H), 3.90 (s, 1 H), 3.87 (s, 3 H), 2.46 (s, 3 H), 2.05-1.94 (m, 1 H), 1. 53 (d, *J* = 12.0 Hz, 1 H), 1.30 (d, *J* = 8.0 Hz, 3 H). ^{13}C NMR (100 MHz, DMSO-d_6): 174.3, 162.2,

155.6, 153.2, 147.4, 143.5, 140.1, 139.5, 137.0, 131.5, 130.8, 130.2, 130.2, 129.7, 121.2, 120.8, 120.2, 119.9, 118.1, 117.5, 116.8, 76.3, 62.2, 60.7, 52.8, 44.8, 29.6, 21.4, 16.2。

Compound 5g：Pure 97.2 %. white solid, – MS (ESI): *m/z* (%) = 545 $[M + H]^+$; ^1H NMR (400 MHz, DMSO-d_6): 12.63 (s, 1 H), 9.36 (s, 1 H), 8.71 (s, 1 H), 8.17 (s, 1 H), 8.03 (dd, J_1 = 4.0 Hz, J_2 = 4.0 Hz, 2 H), 7.87 (d, J = 8.0 Hz, 1 H), 7.69 (d, J = 8.0 Hz, 1 H), 7.50 (t, J = 8.0 Hz, 3 H), 5.39 (s, 1 H), 4.79 (t, J = 4.0 Hz, 1 H), 4.62 (d, J = 12.0 Hz, 1 H), 4.42 (dd, J_1 = 8.0 Hz, J_2 = 8.0 Hz, 1 H), 3.98 (t, J = 12.0 Hz, 1 H), 3.89 (s, 1 H), 3.86 (s, 3 H), 2.01-1.94 (m, 1 H), 1.52 (d, J = 12.0 Hz, 1 H), 1.29 (d, J = 8.0 Hz, 1 H). ^{13}C NMR (100 MHz, DMSO-d_6): 174.3, 162.2, 155.6, 153.2, 147.5, 143.5, 139.5, 131.5, 130.8, 130.3, 122.8, 122.7, 121.3, 120.6, 120.0, 118.1, 117.3, 117.1, 116.8, 76.3, 62.2, 60.7, 52.9, 44.8, 29.6, 16.2。

Compound 5h：Pure 95.9%. white solid, – MS (ESI): *m/z* (%) = 571 $[M + H]^+$; ^1H NMR (400 MHz, DMSO-d_6): 12.62 (s, 1 H), 9.26 (s, 1 H), 8.71 (s, 1 H), 8.16 (s, 1 H), 7.86 (d, J = 8.0 Hz, 3 H), 7.68 (d, J = 8.0 Hz, 1 H),7.48 (t, J = 8.0 Hz, 1 H), 7.15 (d, J = 8.0 Hz, 2 H), 5.39 (t, J = 4.0 Hz, 1 H), 4.79 (t, J = 4.0 Hz, 1 H), 4.62 (d, J = 12.0 Hz, 1 H), 4.42 (dd, J_1 = 8.0 Hz, J_2 = 8.0 Hz, 1 H), 4.13 (dd, J_1 = 4.0 Hz, J_2 = 8.0 Hz, 2 H), 3.98 (t, J = 12.0 Hz, 1 H), 3.89 (s, 1 H), 3.86 (s, 3 H), 2.02-1.93 (m, 1 H), 1.53 (d, J = 12.0 Hz, 1 H), 1.36 (t, J = 8.0 Hz, 3 H), 1.29 (d, J = 8.0 Hz, 3 H). ^{13}C NMR (100 MHz, DMSO-d_6): 173.7, 161.6, 158.5, 155.1, 152.7, 146.7, 143.0, 138.9, 131.1, 130.3, 129.9, 129.7, 121.5, 120.7, 119.7, 119.3, 117.6, 116.2, 115.2, 75.8, 63.5, 61.7, 52.3, 44.3, 29.1, 15.6, 14.5。

Compound 5i：Pure 94.7%. white solid, – MS (ESI): *m/z* (%) = 611 $[M + H]^+$; ^1H NMR (400 MHz, DMSO-d_6): 12.67 (s, 1 H), 9.19 (s, 1 H), 8.77 (s, 1 H), 8.29 (s, 1 H), 7.98 (d, J = 8.0 Hz, 1 H), 7.89 (d, J = 8.0 Hz, 1 H),7.83-7.81 (m, 2 H), 7.76-7.72 (m, 2 H), 7.56 (t, J = 8.0Hz, 1 H), 5.46-5.44 (m, 1 H), 4.88-4.83 (m, 1 H), 4.71-4.66 (m, 1 H), 4.50-4.45 (m, 1 H), 4.04 (t, J = 12.0 Hz, 1 H), 3.96 (s, 1 H), 3.92 (s, 3 H), 2.09-1.99 (m, 1 H), 1.59 (d, J = 16.0 Hz, 1 H), 1.35 (d, J = 8.0 Hz, 3 H). ^{13}C NMR (100 MHz, DMSO-d_6):174.2, 162.2, 155.6, 153.2, 146.9, 143.6, 139.5, 132.1, 131.3, 130.8, 130.3, 129.3, 128.0, 123.9, 123.0, 121.4, 120.0, 118.1, 116.9, 76.3, 62.2, 60.7, 52.8, 44.8, 30.9, 29.6, 16.2。

Compound 5j：Pure 96.6%. white solid, – MS (ESI): *m/z* (%) = 587 $[M + H]^+$; ^1H NMR (400 MHz, DMSO-d_6): 12.62 (s, 1 H), 9.28 (s, 1 H), 8.72 (s, 1 H), 8.16 (s, 1 H), 7.86 (d, J = 8.0 Hz, 1 H), 7.69 (d, J = 8.0 Hz, 1 H), 7.55 (s, 1 H), 7.50 (d, J = 8.0 Hz,

2 H), 7.17 (d, J = 8.0 Hz, 1 H), 5.39 (s, 1 H), 4.79 (t, J = 4.0 Hz, 1 H), 4.62 (d, J = 12.0 Hz, 1 H), 4.42 (t, J = 4.0 Hz, 1 H), 3.98 (t, J = 12.0 Hz,1 H), 3.90 (s, 3 H), 3.85 (d, J = 8.0 Hz, 6 H), 2.01-1.94 (m, 1 H), 1.52 (d, J = 12.0 Hz, 1 H), 1.29 (d, J = 8.0 Hz, 3 H). ^{13}C NMR (100 MHz, DMSO-d_6): 173.7, 161.6, 155.1, 152.7, 149.3, 148.8, 146.7, 143.0, 138.9, 131.1, 130.3, 130.0, 129.7, 120.7, 119.8, 119.3, 117.6, 116.2, 112.0, 104.4, 75.8, 61.7, 60.2, 55.8, 55.7, 52.3, 44.3, 29.1, 15.6。

Compound 5k：Pure 98.7%. white solid, – MS (ESI): m/z (%) = 555 [M + H]$^+$; ^{1}H NMR (400 MHz, DMSO-d_6): 12.61 (s, 1 H), 9.01 (s, 1 H), 8.72 (s, 1 H), 8.22 (s, 1 H), 7.84 (d, J = 8.0Hz, 1 H), 7.69 (d, J = 8.0Hz, 1 H), 7.56 (dd, J_1 = 8.0 Hz, J_2 = 8.0 Hz, 1 H), 7.51-7.44 (m, 3 H), 5.39 (s, 1 H), 4.79 (t, J = 8.0 Hz, 1 H), 4.63 (d, J = 12.0 Hz, 1 H), 4.44-4.39 (m, 1 H), 3.98 (t, J = 12.0 Hz, 1 H), 3.89 (s, 1 H), 3.86 (s, 3 H), 2.55 (d, J = 8.0 Hz, 2 H), 2.01-1.95 (m, 1 H), 1.54 (d, J = 16.0 Hz, 1 H), 1.30 (d, J = 4.0 Hz, 3 H), 1.06 (t, J = 8.0 Hz, 3 H)； ^{13}C NMR (100 MHz, DMSO-d_6): 173.7, 161.6, 155.1, 152.7, 146.1, 143.0, 139.2, 138.9, 135.7, 131.1, 130.3, 130.2, 129.8, 126.9, 126.3, 123.5, 120.8, 119.3, 61.7, 60.2, 52.3, 44.3, 29.1, 23.7, 15.6, 14.8。

Compound 5l：Pure 96.4%. white solid, – MS (ESI): m/z (%) = 557 [M + H]$^+$; ^{1}H NMR (400 MHz, DMSO-d_6): 12.64 (s, 1 H), 9.40 (s, 1 H), 8.72 (s, 1 H), 8.17 (s, 1 H), 7.88 (d, J = 8.0 Hz, 1 H), 7.70 (d, J = 8.0 Hz, 1 H), 7.59-7.48 (m, 4 H), 7.09 (d, J = 8.0 Hz, 1 H), 5.40 (s, 1 H), 4.79 (t, J = 8.0 Hz, 1 H), 4.63 (d, J = 12.0 Hz, 1 H), 4.42 (dd, J_1 = 4.0 Hz, J_2 = 4.0 Hz, 1 H), 3.99 (t, J = 8.0 Hz, 1 H), 3.87 (d, J = 12.0 Hz, 7 H), 2.03-1.94 (m, 1 H), 1.53 (d, J = 16.0 Hz, 1 H), 1.30 (d, J = 4.0 Hz, 3 H); ^{13}C NMR (100 MHz, DMSO-d_6): 173.7, 161.6, 160.2, 155.1, 152.7, 146.9, 143.0, 138.9, 137.6, 130.9, 130.8, 130.3, 129.7, 120.7, 119.8, 119.4, 117.6, 116.3, 114.4, 111.8, 105.5, 75.8, 61.7, 60.2, 55.6, 52.3, 44.3, 29.1, 15.6。

Compound 5m：Pure 95.7%. white solid, – MS (ESI): m/z (%) = 555 [M + H]$^+$; ^{1}H NMR (400 MHz, DMSO-d_6): 12.63 (s, 1 H), 9.34 (s, 1 H), 8.73 (s, 1 H), 8.17 (s, 1 H), 7.89 (t, J = 8.0 Hz, 3 H), 7.68 (dd, J_1 = 8.0 Hz, J_2 = 8.0 Hz, 3 H), 7.50 (t, J = 8.0 Hz, 1 H), 5.40 (s, 1 H), 4.79 (t, J = 8.0 Hz,1 H), 4.63 (d, J = 12.0 Hz, 1 H), 4.43 (dd, J_1 = 4.0 Hz, J_2 = 4.0 Hz, 1 H), 3.99 (t, J = 8.0 Hz, 1 H), 3.90 (s, 1 H), 3.86 (s, 3 H), 2.03-1.94 (m, 1 H), 1.53 (d, J = 16.0 Hz, 1 H), 1.35 (s, 8 H), 1.30 (d, J = 4.0 Hz, 3H); ^{13}C NMR (100 MHz, DMSO-d_6): 173.7, 161.6, 155.1, 152.7, 151.3, 146.8, 143.0, 138.9, 134.3, 131.1, 126.6, 120.7, 119.7, 119.6, 119.4, 117.6, 116.3, 75.8, 61.7, 60.2, 52.3, 44.3, 34.4, 30.9, 29.1, 15.6。

Compound 5n：Pure 97.6%. white solid, – MS (ESI): m/z (%) = 583 [M + H]$^+$; ^{1}H

NMR (400 MHz, DMSO-d_6): 12.66 (s, 1 H), 9.35 (s, 1 H), 8.68 (s, 1 H), 8.17 (s, 1 H), 7.89 (t, *J* = 8.0 Hz, 3 H), 7.68 (dd, J_1 = 8.0 Hz, J_2 = 8.0 Hz, 3 H), 7.50 (t, *J* = 8.0 Hz, 1 H), 5.47-5.39 (m, 1 H), 4.74-4.62 (m, 1 H), 4.28 (m, 1 H), 4.12-3.96 (m, 3 H), 3.89-3.86 (m, 3 H), 2.28-2.19 (m, 1 H), 2.00-1.88 (m, 1 H), 1.41 (d, *J* = 4.0Hz, 3 H), 1.35 (s, 9 H); ^{13}C NMR (100 MHz, DMSO-d_6): 174.2, 162.2, 154.6, 153.2, 152.2, 151.8, 147.3, 143.6, 139.4, 134.8, 131.6, 130.2, 127.1, 121.2, 120.1, 117.9, 116.8, 77.5, 76.3, 61.2, 60.7, 54.4, 52.8, 45.4, 35.0, 31.4, 20.2, 16.1。

Compound 5o：Pure 99.1%. white solid, – MS (ESI): *m/z* (%) = 527 $[M + H]^+$; ^{1}H NMR (400 MHz, $CDCl_3$): 12.37 (s, 1 H), 8.46 (s, 1 H), 8.26 (s, 2 H), 7.81 (d, *J* = 8.0 Hz, 2 H), 7.71 (d, *J* = 8.0 Hz, 1 H), 7.55 (t, *J* = 8.0 Hz, 2 H), 7.45-7.42 (m, 2 H), 5.23 (s, 1 H), 5.00 (t, *J* = 8.0 Hz, 1 H), 4.34-4.31 (m, 1 H), 4.22-4.17 (m, 1 H), 4.08 (s, 3 H), 3.95 (d, *J* = 8.0 Hz, 2 H), 2.23-2.13 (m, 1 H), 1.67 (s, 1 H), 1.53-1.50 (m, 1 H), 1.35 (d, *J* = 8.0 Hz, 3 H); ^{13}C NMR (100 MHz, $CDCl_3$): 174.6, 161.8, 155.5, 154.6, 148.1, 142.2, 138.9, 137.1, 131.0, 129.7, 129.6, 129.3, 128.7, 121.6, 120.4, 120.4, 119.2, 117.9, 117.7, 76.1, 62.5, 61.2, 53.6, 44.6, 29.4, 16.0。

Compound 5p：Pure 98.9%. yellow solid, – MS (ESI): *m/z* (%) = 595 $[M + H]^+$; ^{1}H NMR (400 MHz, DMSO-d_6): 12.67 (s, 1 H), 9.58 (s, 1 H), 8.73 (s, 1 H), 8.37-8.35 (m, 2 H), 8.19 (s, 1 H), 7.91-7.88 (m, 3 H), 7.71 (d, *J* = 8.0 Hz, 1 H), 7.52 (t, *J* = 8.0 Hz, 1 H), 5.40 (t, *J* = 4.0 Hz, 1 H), 4.79 (t, *J* = 8.0 Hz,1 H), 4.66-4.62 (m, 1 H), 4.45-4.40 (m, 1 H), 3.99 (t, *J* = 12.0 Hz, 1 H), 3.90 (d, *J* = 8.0 Hz, 1 H), 3.86 (s, 3 H), 2.03-1.93 (m, 1 H), 1.55-1.51 (m, 1 H), 1.30 (d, *J* = 4.0 Hz, 3 H); ^{13}C NMR (100 MHz, DMSO-d_6): 174.2, 162.2, 155.6, 153.2, 147.7, 143.5, 139.5, 137.5, 131.8, 131.2, 130.8, 130.3, 125.7, 125.4, 124.3, 122.7, 11.2, 120.6, 120.0, 118.1, 117.0, 116.8, 76.3, 62.2, 60.7, 52.8, 44.8, 29.6, 16.1。

Compound 5q：Pure 98.3%. yellow solid, – MS (ESI): *m/z* (%) = 663 $[M + H]^+$; ^{1}H NMR (400 MHz, DMSO-d_6): 12.65 (s, 1 H), 9.19 (s, 1 H), 8.73 (s, 1 H), 8.45 (s, 1 H), 8.35-8.29 (m, 2 H), 8.25 (s, 1 H), 7.83 (d, *J* = 8.0 Hz, 1 H), 7.69 (d, *J* = 8.0 Hz, 1 H), 7.51 (t, *J* = 8.0 Hz, 1 H), 5.40 (t, *J* = 4.0 Hz, 1 H), 4.79 (t, *J* = 8.0 Hz, 1 H), 4.66-4.61 (m, 1 H), 4.45-4.40 (m, 1 H), 3.99 (t, *J* = 12.0 Hz, 1 H), 3.90 (s, 1 H), 3.85 (s, 3 H), 2.02-1.93 (m, 1 H), 1.55-1.51 (m, 1 H), 1.29 (d, *J* = 4.0 Hz, 3 H); ^{13}C NMR (100 MHz, DMSO-d_6): 174.2, 162.2, 155.6, 153.2, 146.8, 143.5, 139.5, 135.6, 134.5, 134.2, 131.1, 130.8, 130.3, 129.7, 128.6, 127.1, 125.1, 124.5, 123.9, 121.3, 120.1, 118.1, 116.9, 76.3, 62.2, 60.6, 52.8, 44.8, 29.6, 16.1。

Compound 5r：Pure 97.7%. yellow solid, – MS (ESI): *m/z* (%) = 663 $[M + H]^+$; ^{1}H

NMR (400 MHz, DMSO-d_6): 12.68 (s, 1 H), 9.69 (s, 1 H), 8.72 (d, *J* = 4.0 Hz, 3 H), 8.31 (s, 1 H), 8.19 (m, 2 H), 7.88 (d, *J* = 8.0 Hz, 1 H), 7.71 (d, *J* = 8.0 Hz, 1 H), 7.53 (t, *J* = 8.0 Hz, 1 H), 5.42-5.39 (m, 1 H), 4.79 (t, *J* = 8.0 Hz, 1 H), 4.66-4.62 (m, 1 H), 4.45-4.40 (m, 1 H), 3.99 (t, *J* = 12.0 Hz, 1 H), 3.91-3.90 (m, 1 H), 3.86 (s, 3 H), 2.03-1.94 (m, 1 H), 1.55-1.51 (m, 1 H), 1.30 (d, *J* = 8.0 Hz, 3 H); ^{13}C NMR (100 MHz, DMSO-d_6): 174.3, 162.2, 155.6, 153.2, 150.2, 147.9, 143.5, 139.5, 138.3, 132.1, 131.0, 130.9, 130.4, 124.6, 121.2, 121.1, 121.0, 120.2, 118.0, 116.8, 76.3, 62.2, 60.7, 52.8, 44.8, 29.6, 16.1。

Compound 5s：Pure 95.3%. white solid, – MS (ESI): *m/z* (%) = 541 $[M + H]^+$; ^{1}H NMR (400 MHz, DMSO-d_6): 12.62 (s, 1 H), 9.03 (s, 1 H), 8.73 (s, 1 H), 8.21 (s, 1 H), 7.85 (d, *J* = 8.0 Hz, 1 H), 7.69 (d, *J* = 8.0 Hz, 1 H), 7.55-7.43 (m, 5 H), 5.41-5.38 (m, 1 H), 4.82-4.76 (m, 1 H), 4.65-4.61 (m, 1 H), 4.44-4.39 (m, 1 H), 3.98 (t, *J* = 12.0 Hz, 1 H), 3.90-3.88 (m, 1 H), 3.85 (s, 3 H), 2.25 (s, 3 H), 2.02-1.93 (m, 1 H), 1.54-1.51 (m, 1 H), 1.29 (d, *J* = 8.0 Hz, 3 H); ^{13}C NMR (100 MHz, DMSO-d_6): 174.2, 162.1, 155.6, 153.2, 146.6, 143.6, 139.4, 136.7, 133.4, 131.9, 131.7, 130.8, 130.3, 130.2, 127.5, 126.4, 123.8, 121.3, 119.8, 118.1, 116.8, 76.3, 62.2, 60.7, 52.8, 44.8, 29.6, 18.0, 16.1。

Compound 5t：Pure 98.1%. white solid, – MS (ESI): *m/z* (%) = 557 $[M + H]^+$; ^{1}H NMR (400 MHz, DMSO-d_6): 12.62 (s, 1 H), 8.98 (s, 1 H), 8.68 (s, 1 H), 8.21 (s, 1 H), 7.82-7.81 (m, 1 H), 7.69 (d, *J* = 8.0 Hz, 2 H), 7.59-7.55 (m, 1 H), 7.48 (t, *J* = 8.0 Hz, 1 H), 7.39 (d, *J* = 8.0 Hz, 1 H), 7.18 (t, *J* = 8.0 Hz, 1 H), 5.46-5.39 (m, 1 H), 4.73-4.61 (m, 1 H), 4.31-4.24 (m, 1 H), 4.09-3.99 (m, 3 H), 3.90 (s, 3 H), 3.87 (d, *J* = 8.0 Hz, 3 H), 2.29-2.18 (s, 1 H), 1.99-1.88 (m, 1 H), 1.41-1.28 (m, 3 H); ^{13}C NMR (100 MHz, $CDCl_3$): 161.7, 151.1, 146.9, 142.1, 141.9, 138.7, 131.4, 130.0, 129.5, 126.4, 125.5, 122.0, 121.7, 121.2, 120.2, 119.3, 117.6, 112.3, 99.9, 61.3, 61.2, 56.1, 55.2, 45.4, 26.6, 19.9。

Compound 5u：Pure 97.7%. white solid, – MS (ESI): *m/z* (%) = 605 $[M + H]^+$; ^{1}H NMR (600 MHz, DMSO-d_6): 12.61 (s, 1 H), 9.09 (s, 1 H), 8.71 (s, 1 H), 8.25-8.21 (m, 1 H), 7.98 (dd, J_1 = 6.0 Hz, J_2 = 12.0 Hz, 1 H), 7.87-7.48 (m, 6 H), 5.40 (d, *J* = 12.0 Hz, 1 H), 4.79 (t, *J* = 6.0 Hz, 1 H), 4.64-4.61 (m, 1 H), 4.42 (dd, J_1 = 6.0 Hz, J_2 = 6.0 Hz, 1 H), 3.98 (t, *J* = 12.0 Hz, 1 H), 3.90-3.88 (m, 1 H), 3.86 (s, 3 H), 2.01-1.95 (m, 1 H), 1.54-1.52 (m, 1 H), 1.29 (d, *J* = 6.0 Hz, 3 H).^{13}C NMR (150 MHz, DMSO-d_6): 174.2, 162.2, 155.6, 153.2, 146.6, 143.5, 139.4, 136.6, 134.1, 132.5, 131.4, 130.8, 130.4, 129.5, 129.1, 124.3, 120.4, 120.0, 119.3, 118.1, 116.8, 76.3, 62.2, 60.7, 52.8, 44.8, 29.6, 16.1。

Compound 5v: Pure 96.4%. white solid, – MS (ESI): *m/z* (%) = 561 $[M + H]^+$; ^1H NMR (400 MHz, DMSO-d_6): 12.65-12.63 (m, 1 H), 9.13 (s, 1 H), 8.73-8.68 (m, 1 H), 8.23 (s, 1 H), 7.84-7.79 (m, 3 H), 7.70-7.61 (m, 3 H), 7.52-7.42 (m, 2 H), 5.46-5.39 (m, 1 H), 4.74-4.61 (m, 1 H), 4.30-4.25 (m, 1 H), 4.09-3.99 (m, 2 H), 3.88-3.85 (m, 3 H), 2.29-2.16 (m, 1 H), 1.98-1.88 (m, 1 H), 1.41-1.28 (m, 3 H); ^{13}C NMR (100 MHz, DMSO-d_6): 174.2, 162.2, 155.6, 154.6, 153.2, 152.2, 146.7, 143.6, 139.4, 134.9, 132.2, 131.0, 130.3, 129.0, 126.6, 124.3, 121.3, 120.0, 117.9, 116.9, 77.5, 60.7, 52.8, 45.4, 29.6, 26.4, 20.2, 16.1。

Compound 5w: Pure 98.2%. white solid, – MS (ESI): *m/z* (%) = 653 $[M + H]^+$; ^1H NMR (400 MHz, DMSO-d_6): 12.65-12.63 (m, 1 H), 9.41 (s, 1 H), 8.73 (s, 1 H), 8.18 (s, 1 H), 7.99 (d, J = 8.0 Hz, 1 H), 7.90-7.83 (m, 1 H), 7.72-7.63 (m, 3 H), 7.55-7.47 (m, 2 H), 5.40 (t, J = 4.0 Hz, 1 H), 4.82-4.76 (m, 1 H), 4.66-4.62 (m, 1 H), 4.45-4.40 (m, 1 H), 4.01-3.96 (m, 1 H), 3.89 (d, J = 8.0 Hz, 1 H), 3.86-3.85 (m, 3 H), 2.01-1.93 (m, 1 H), 1.54-1.51 (m,1 H), 1.30 (d, J = 4.0 Hz, 3 H). ^{13}C NMR (100 MHz, DMSO-d_6): 174.2, 162.2, 155.6, 153.2, 147.5, 143.5, 140.2, 139.5, 137.1, 131.5, 130.8, 130.4, 130.3, 129.2, 121.2, 120.4, 120.3, 119.9, 118.1, 116.8, 76.3, 62.2, 60.7, 55.3, 52.8, 44.8, 29.6, 16.1。

Compound 5x: Pure 94.1%. white solid, – MS (ESI): *m/z* (%) = 557 $[M + H]^+$; ^1H NMR (400 MHz, DMSO-d_6): 12.65-12.63 (m, 1 H), 9.28 (s, 1 H), 8.73-8.68 (m, 1 H), 8.16 (s, 1 H), 7.90-7.85 (m, 3 H), 7.69 (d, J = 8.0 Hz, 1 H), 7.49 (t, J = 8.0 Hz, 1 H), 7.18 (d, J = 8.0 Hz, 2 H), 5.46-5.39 (m, 1 H), 4.82-4.76 (m, 1 H), 4.81-4.28 (m, 3 H), 4.28-4.12 (m, 2 H), 3.99 (s, 3 H), 3.86 (d, J = 4.0Hz, 3 H), 2.34-2.18 (m, 1 H), 2.02-1.88 (m, 1 H), 1.41-1.29 (m, 3 H). ^{13}C NMR (100 MHz, DMSO-d_6): 162.2, 159.7, 154.6, 153.2, 152.2, 147.2, 143.6, 139.4, 131.6, 130.5, 130.2, 122.0, 121.2, 120.3, 117.9, 116.8, 115.4, 77.5, 76.3, 61.2, 60.7, 56.0, 45.4, 29.6, 26.4, 20.2, 16.1。

Compound 5y: Pure 94.9%. white solid, – MS (ESI): *m/z* (%) = 541 $[M + H]^+$; ^1H NMR (400 MHz, DMSO-d_6): 12.64 (s, 1 H), 9.35 (s, 1 H), 8.73 (s, 1 H), 8.17 (s, 1 H), 7.89-7.85 (m, 3 H), 7.69 (d, J = 4.0 Hz, 1 H), 7.52-7.43 (m, 3 H), 5.41-5.39 (m, 1 H), 4.83-4.76 (m, 1 H), 4.66-4.61 (m, 1 H), 4.42 (dd, J_1 = 4.0 Hz, J_2 = 4.0 Hz, 1 H), 4.01-3.95 (m, 1 H), 3.99 (d, J = 8.0 Hz, 1 H), 3.86 (s, 3 H), 2.41 (s, 3 H), 2.02-1.94 (m, 1 H), 1.55-1.51 (m, 1 H), 1.30 (d, J = 4.0 Hz, 3 H). ^{13}C NMR (150 MHz, DMSO-d_6): 174.3, 162.2, 155.6, 153.2, 147.4, 143.5, 139.4, 138.8, 134.8, 131.5, 130.7, 130.3, 120.3, 120.2, 119.9, 118.1, 116.8, 76.3, 62.2, 60.7, 56.5, 52.8, 44.8, 29.6, 21.0, 19.0, 16.1。

Compound 5z: Pure 94.4%. white solid, – MS (ESI): *m/z* (%) = 569 $[M + H]^+$; ^1H NMR (400 MHz, DMSO-d_6): 12.62 (s, 1 H), 8.86 (s, 1 H), 8.72 (s, 1 H), 8.21 (s, 1 H), 7.83 (d, J = 12.0 Hz, 1 H), 7.67 (d, J = 8.0 Hz, 1 H), 7.48 (t, J = 8.0 Hz, 1 H), 7.13 (s, 2 H), 5.41-5.38 (m, 1 H), 4.82-4.75 (m, 1 H), 4.65-4.61 (m, 1 H), 4.42 (dd, J_1 = 4.0 Hz, J_2 = 4.0 Hz, 1 H), 4.01-3.96 (m, 1 H), 3.90-3.87 (m, 1 H), 3.85 (s, 3 H), 2.35 (s, 3 H), 1.96 (s, 6 H), 1.29 (d, J = 8.0 Hz, 3 H). ^{13}C NMR (100 MHz, DMSO-d_6): 174.2, 162.1, 155.6, 153.2, 146.6, 143.5, 140.0, 139.4, 134.9, 133.8, 131.8, 130.8, 130.2, 129.4, 124.1, 121.3, 119.8, 118.1, 116.8, 76.3, 62.2, 60.7, 52.8, 44.8, 29.6, 21.1, 17.3, 16.1。

Compound 6a: Pure 98.6%. white solid, – MS (ESI): *m/z* (%) = 697 $[M + H]^+$; ^1H NMR (400 MHz, DMSO-d_6): 12.59 (s, 1 H), 8.76 (s, 1 H), 8.71 (s, 1 H), 8.15 (s, 1 H), 7.86 (d, J = 4.0 Hz, 1 H), 7.77 (d, J = 12.0 Hz, 1 H), 7.64-7.59 (m, 3 H), 7.44 (t, J = 8.0 Hz, 1 H), 5.68 (s, 2 H), 5.41-5.38 (m, 1 H), 4.80-4.76 (m, 1 H), 4.65-4.60 (m, 1 H), 4.41 (dd, J_1 = 8.0 Hz, J_2 = 4.0 Hz, 1 H), 4.01-3.95 (m, 1 H), 3.90-3.89 (m, 1 H), 3.85 (s, 3 H), 2.01-1.93 (m, 1 H), 1.54-1.51 (m, 1 H), 1.29 (d, J = 8.0 Hz, 3 H). ^{13}C NMR (100 MHz, DMSO-d_6): 174.2, 162.1, 155.6, 153.2, 146.9, 143.5, 140.7, 139.4, 133.7, 131.7, 130.8, 130.7, 130.2, 123.2, 122.6, 121.1, 119.7, 118.1, 116.7, 76.3, 62.2, 60.7, 52.8, 52.0, 44.8, 29.6, 16.1。

Compound 6b: Pure 99.2%. white solid, – MS (ESI): *m/z* (%) = 541 $[M + H]^+$; ^1H NMR (400 MHz, DMSO-d_6): 12.58 (s, 1 H), 8.72 (d, J = 4.0 Hz, 1 H), 8.13 (s, 1 H), 7.76 (d, J = 8.0 Hz, 1 H), 7.59 (d, J = 4.0 Hz, 1 H), 7.45-7.35 (m, 6 H), 5.66 (s, 2 H), 5.40-5.38 (m, 1 H), 4.82-4.75 (m, 1 H), 4.65-4.60 (m, 1 H), 4.41 (dd, J_1 = 4.0 Hz, J_2 = 4.0 Hz, 1 H), 4.03-3.98 (m, 1 H), 3.90-3.89 (m, 1 H), 3.85 (s, 3 H), 2.02-1.93 (m, 1 H), 1.54-1.50 (m, 1 H), 1.29 (d, J = 8.0 Hz, 3 H). ^{13}C NMR (100 MHz, DMSO-d_6): 174.2, 162.1, 155.6, 153.2, 146.8, 143.5, 139.3, 136.4, 131.9, 130.8, 130.1, 129.2, 128.6, 128.4, 122.3, 121.1, 119.6, 118.1, 116.6, 76.3, 62.2, 60.7, 53.5, 52.8, 44.8, 29.6。

Compound 6c: Pure 97.8%. white solid, – MS (ESI): *m/z* (%) = 619 $[M + H]^+$; ^1H NMR (400 MHz, DMSO-d_6): 12.58 (s, 1 H), 8.71 (s, 1 H), 8.68 (s, 1 H), 8.15 (s, 1 H), 7.77-7.71 (m, 2 H), 7.61 (d, J = 4.0 Hz, 1 H), 7.46-7.42 (m, 2 H), 7.36-7.32 (m, 1 H), 7.27 (d, J = 4.0 Hz, 1 H), 5.75 (s, 2 H), 5.40-5.38 (m, 1 H), 4.82-4.77 (m, 1 H), 4.65-4.60 (m, 1 H), 4.41 (dd, J_1 = 4.0 Hz, J_2 = 8.0 Hz, 1 H), 4.01-3.95 (m, 1 H), 3.90-3.89 (m, 1 H), 3.85 (s, 3 H), 2.01-1.93 (m, 1 H), 1.54-1.50 (m, 1 H), 1.29 (d, J = 8.0 Hz, 3 H). ^{13}C NMR (100 MHz, DMSO-d_6): 174.2, 162.1, 155.6, 153.6, 153.2, 146.6, 143.5, 139.3, 135.2, 133.4, 131.8, 131.0, 130.9, 130.8, 130.1, 128.8, 123.3, 122.7, 121.2, 119.6, 118.1, 116.7, 76.3, 62.2, 60.7, 60.5, 53.6, 52.8, 44.8, 29.6, 16.1。

Compound 6d: Pure 96.9%. white solid, – MS (ESI): *m/z* (%) = 571 $[M + H]^+$; ^1H NMR (400 MHz, DMSO-d_6): 12.57 (s, 1 H), 8.70 (s, 1 H), 8.13 (s, 1 H), 7.76 (d, J = 8.0 Hz, 1 H), 7.59 (d, J = 8.0 Hz, 1 H), 7.43 (t, J = 8.0 Hz, 1 H), 7.31 (t, J = 8.0 Hz, 1 H), 6.97-6.91 (m, 3 H), 5.61 (s, 2 H), 5.40-5.38 (m, 1 H), 4.83-4.75 (m, 1 H), 4.64-4.60 (m, 1 H), 4.41 (dd, J_1 = 4.0 Hz, J_2 = 8.0 Hz, 1 H), 4.01-3.95 (m, 1 H), 3.90-3.89 (m, 1 H), 3.85 (s, 3 H), 3.76 (s, 3 H), 2.02-1.93 (m, 1 H), 1.54-1.51 (m, 1 H), 1.29 (d, J = 8.0 Hz, 3 H). ^{13}C NMR (100 MHz, DMSO-d_6): 174.2, 162.1, 159.9, 155.6, 153.2, 146.8, 143.5, 139.3, 137.8, 131.9, 130.8, 130.4, 130.1, 122.3, 121.1, 120.5, 119.6, 118.1, 116.6, 114.2, 114.0, 76.3, 62.2, 60.7, 55.6, 53.4, 52.8, 44.8, 29.6, 16.1。

Compound 6e: Pure 98.9%. white solid, – MS (ESI): *m/z* (%) = 619 $[M + H]^+$; ^1H NMR (400 MHz, DMSO-d_6): 12.59 (s, 1 H), 8.75 (s, 1 H), 8.71 (s, 1 H), 8.14 (s, 1 H), 7.76 (d, J = 8.0 Hz, 1 H), 7.63-7.55 (m, 3 H), 7.44 (t, J = 8.0 Hz, 1 H), 7.38 (d, J = 4.0 Hz, 2 H), 5.68 (s, 2 H), 5.41-5.38 (m, 1 H), 4.80-4.77 (m, 1 H), 4.65-4.60 (m, 1 H), 4.41 (dd, J_1 = 4.0 Hz, J_2 = 4.0 Hz, 1 H), 4.01-3.95 (m, 1 H), 3.90-3.89 (m, 1 H), 3.85 (s, 3 H), 2.02-1.93 (m, 1 H), 1.54-1.51 (m, 1 H), 1.29 (d, J = 8.0 Hz, 3 H). ^{13}C NMR (100 MHz, DMSO-d_6): 174.2, 162.1, 155.6, 153.2, 146.9, 143.5, 139.4, 139.0, 131.8, 131.5, 131.5, 131.2, 130.8, 130.2, 127.6, 122.4, 122.3, 121.1, 119.6, 118.1, 116.7, 76.3, 62.2, 60.7, 52.8, 52.7, 44.8, 29.6, 16.1。

Compound 6f: Pure 98.4%. white solid, – MS (ESI): *m/z* (%) = 559 $[M + H]^+$; ^1H NMR (400 MHz, DMSO-d_6): 12.57 (s, 1 H), 8.71 (s, 1 H), 8.69 (s, 1 H), 8.14 (s, 1 H), 7.76 (d, J = 12.0 Hz, 1 H), 7.60 (d, J = 8.0 Hz, 1 H), 7.47-7.40 (m, 3 H), 7.31-7.24 (m, 2 H), 5.72 (s, 2 H), 5.40-5.38 (m, 1 H), 4.82-4.76 (m, 1 H), 4.65-4.60 (m, 1 H), 4.41 (dd, J_1 = 4.0 Hz, J_2 = 8.0 Hz, 1 H), 4.01-3.95 (m, 1 H), 3.90-3.89 (m, 1 H), 3.85 (s, 3 H), 2.02-1.93 (m, 1 H), 1.54-1.51 (m, 1 H), 1.29 (d, J = 8.0 Hz, 3 H). ^{13}C NMR (100 MHz, DMSO-d_6): 174.2, 162.1, 155.6, 153.2, 146.7, 143.5, 139.3, 131.8, 131.2, 130.8, 130.1, 125.4, 123.2, 123.1, 122.4, 121.1, 119.6, 118.1, 116.7, 116.2, 116.0, 76.3, 62.2, 60.7, 52.8, 47.5, 44.8, 29.6, 16.1。

Compound 6g: Pure 97.6%. white solid, – MS (ESI): *m/z* (%) = 555 $[M + H]^+$; ^1H NMR (400 MHz, DMSO-d_6): 12.57 (s, 1 H), 8.71 (s, 1 H), 8.61 (s, 1 H), 8.13 (s, 1 H), 7.76 (d, J = 8.0 Hz, 1 H), 7.60 (d, J = 8.0 Hz, 1 H), 7.43 (t, J = 8.0 Hz, 1 H), 7.27-7.16 (m, 4 H), 5.66 (s, 2 H), 5.40-5.38 (m, 1 H), 4.80-4.77 (m, 1 H), 4.64-4.60 (m, 1 H), 4.41 (dd, J_1 = 4.0 Hz, J_2 = 8.0 Hz, 1 H), 4.01-3.95 (m, 1 H), 3.90-3.89 (m, 1 H), 3.85 (s, 3 H), 2.36 (s, 3 H), 2.02-1.93 (m, 1 H), 1.54-1.51 (m, 1 H), 1.29 (d, J = 8.0 Hz, 3 H). ^{13}C NMR (100 MHz, DMSO-d_6): 174.2, 162.1, 155.6, 153.2, 146.7, 143.5, 139.3,

136.8, 134.5, 131.9, 130.9, 130.1, 129.2, 128.8, 126.8, 122.3, 121.1, 119.6, 118.1, 116.7, 99.9, 76.3, 62.2, 60.7, 52.8, 51.6, 44.8, 29.6, 19.1, 16.1。

Compound 6h：Pure 98.5%. white solid, – MS (ESI): *m/z* (%) = 609 $[M + H]^+$; ^1H NMR (400 MHz, DMSO-d_6): 12.57 (s, 1 H), 8.70 (d, *J* = 4.0 Hz, 2 H), 8.17 (s, 1 H), 7.84 (d, *J* = 8.0 Hz, 1 H), 7.78-7.70 (m, 2 H), 7.61 (t, *J* = 8.0 Hz, 2 H), 7.45 (t, *J* = 8.0 Hz, 1 H), 7.29 (d, *J* = 8.0 Hz, 1 H), 5.86 (s, 2 H), 5.41-5.38 (m, 1 H), 4.83-4.76 (m, 1 H), 4.65-4.61 (m, 1 H), 4.41 (dd, J_1 = 8.0 Hz, J_2 = 4.0 Hz, 1 H), 4.021-3.96 (m, 1 H), 3.90-3.88 (m, 1 H), 3.86 (s, 3 H), 2.03-1.94 (m, 1 H), 1.55-1.51 (m, 1 H), 1.30 (d, *J* = 8.0 Hz, 3 H). ^{13}C NMR (100 MHz, DMSO-d_6):174.2, 162.1, 155.6, 153.2, 146.8, 143.5, 139.4, 134.0, 133.7, 131.8, 130.8, 130.1, 129.4, 136.6, 122.8, 121.2, 119.7, 118.1, 116.8, 76.3, 62.2, 60.7, 52.9, 50.2, 44.8, 29.6, 16.1。

Figure S1. ^{1}H NMR and ^{13}C NMR spectrums of compound 5a

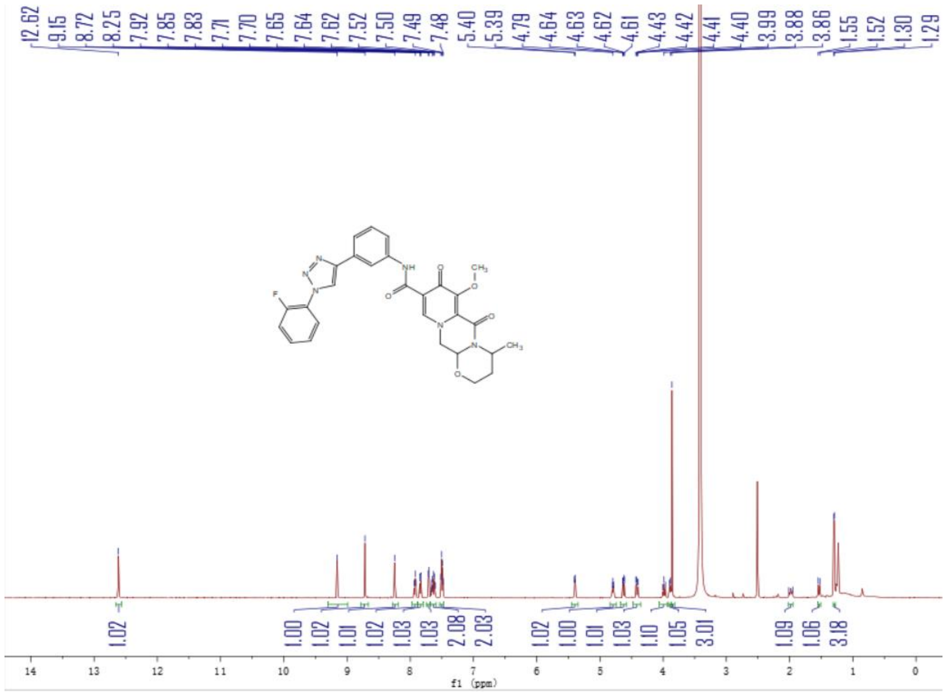

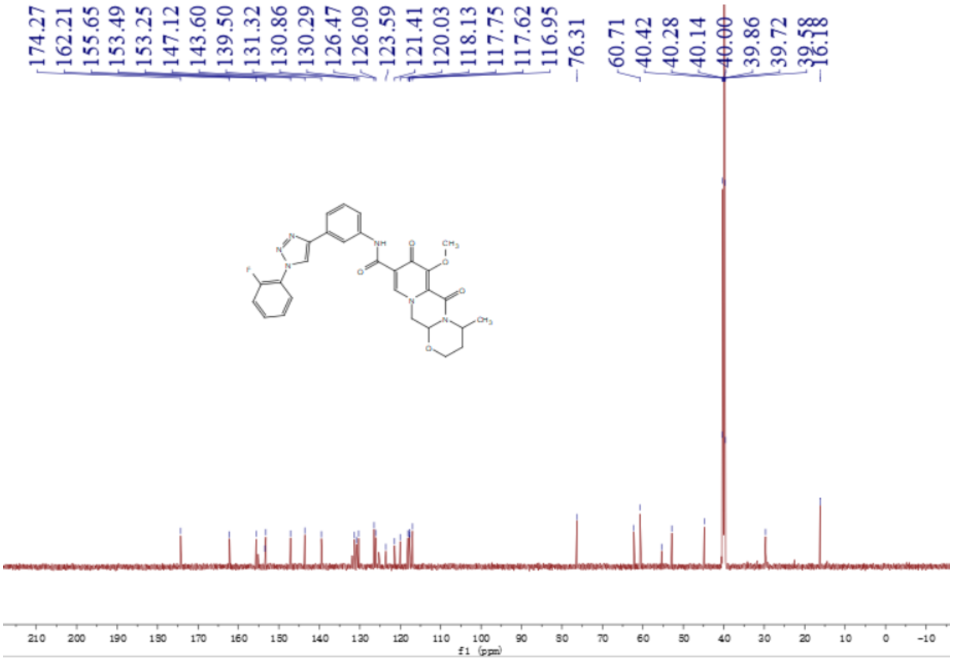

Figure S2. ^{1}H NMR and ^{13}C NMR spectrums of compound 5b

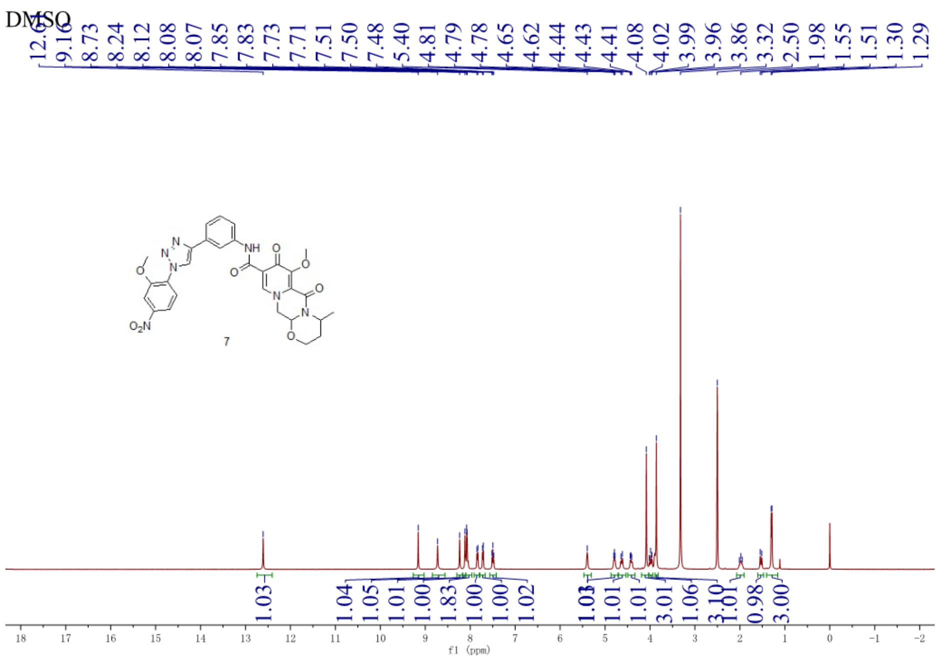

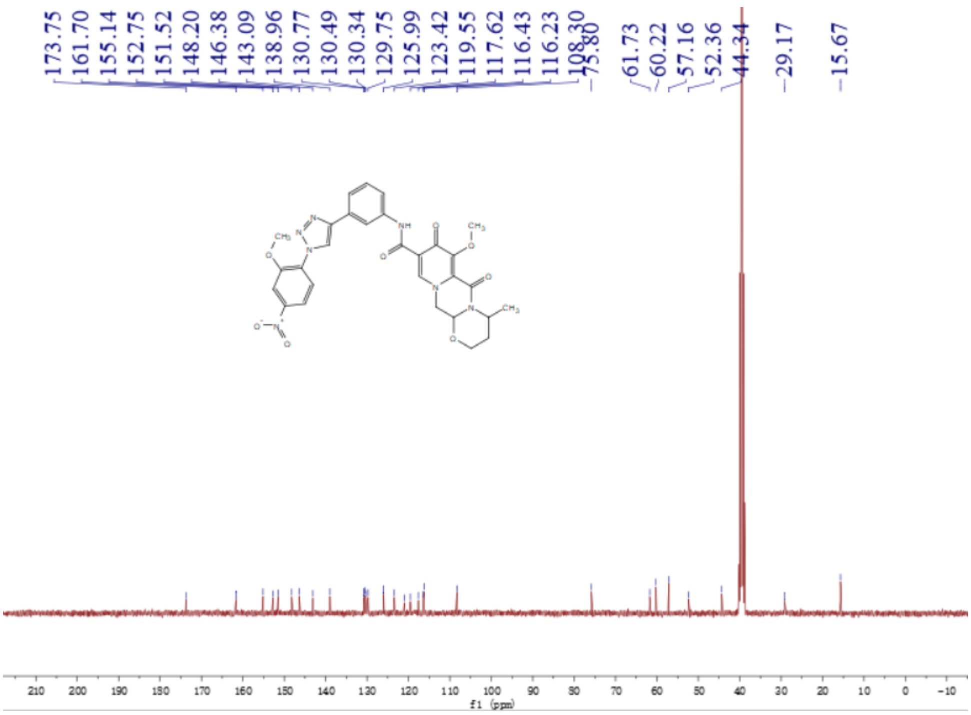

Figure S3. ^{1}H NMR and ^{13}C NMR spectrums of compound 5c

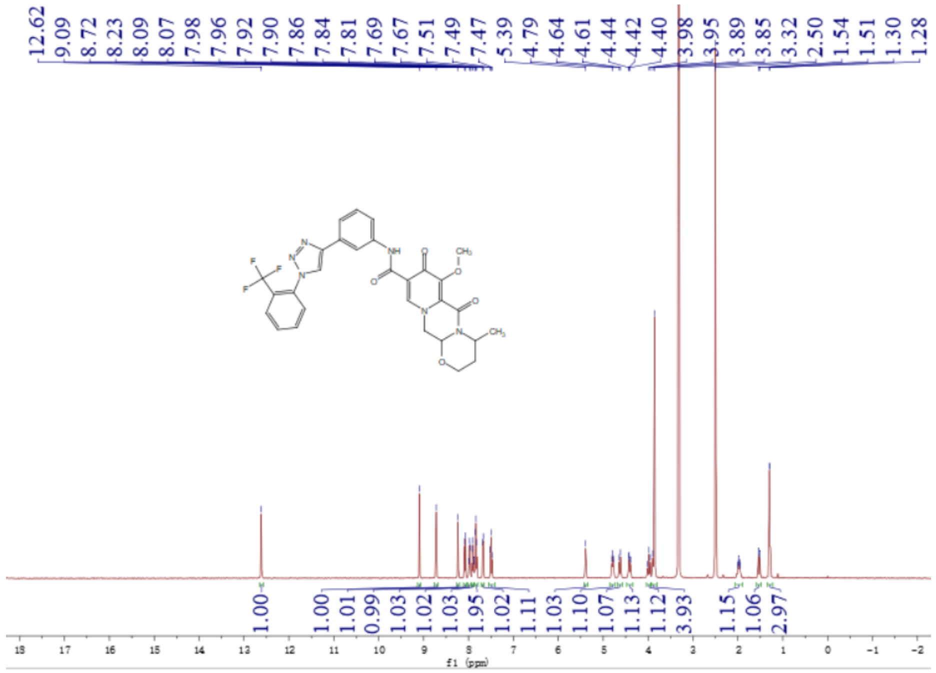

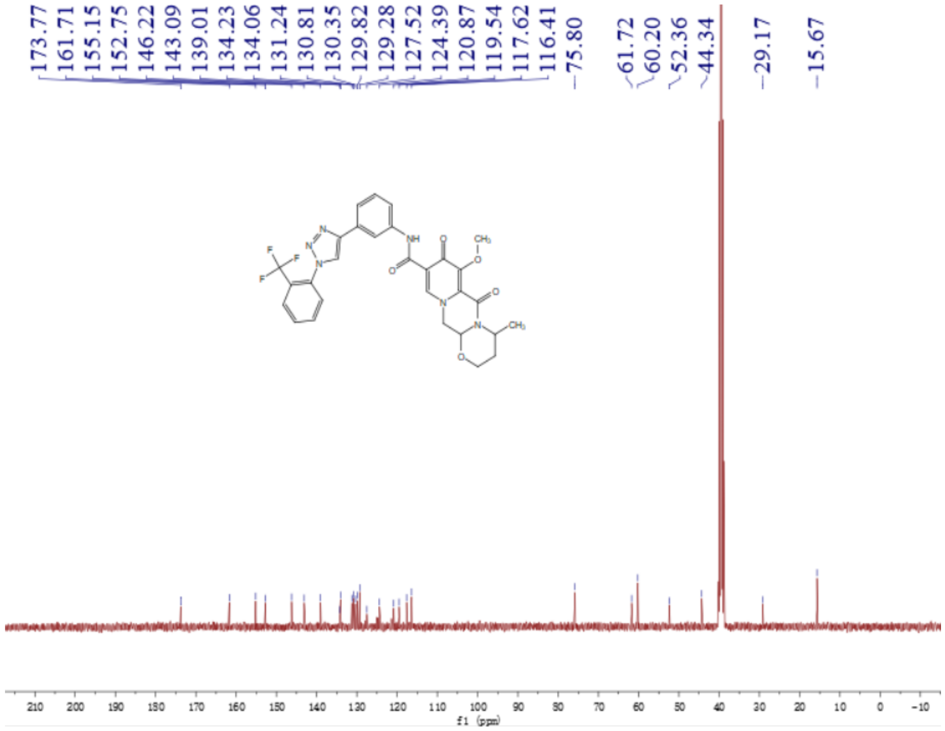

Figure S4. ^{1}H NMR and ^{13}C NMR spectrums of compound 5d

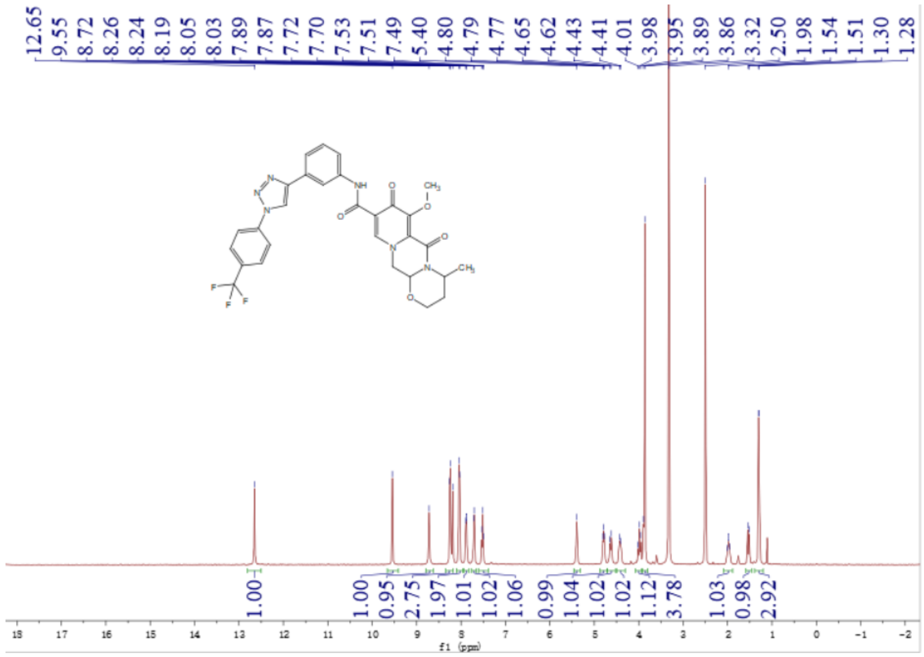

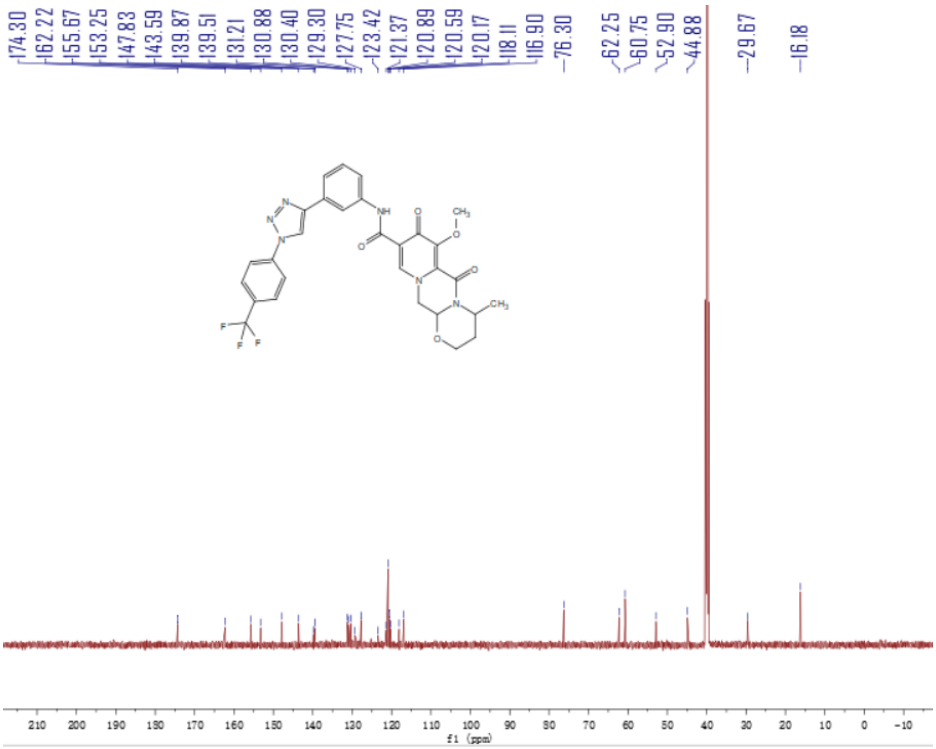

Figure S5. ^{1}H NMR and ^{13}C NMR spectrums of compound 5e

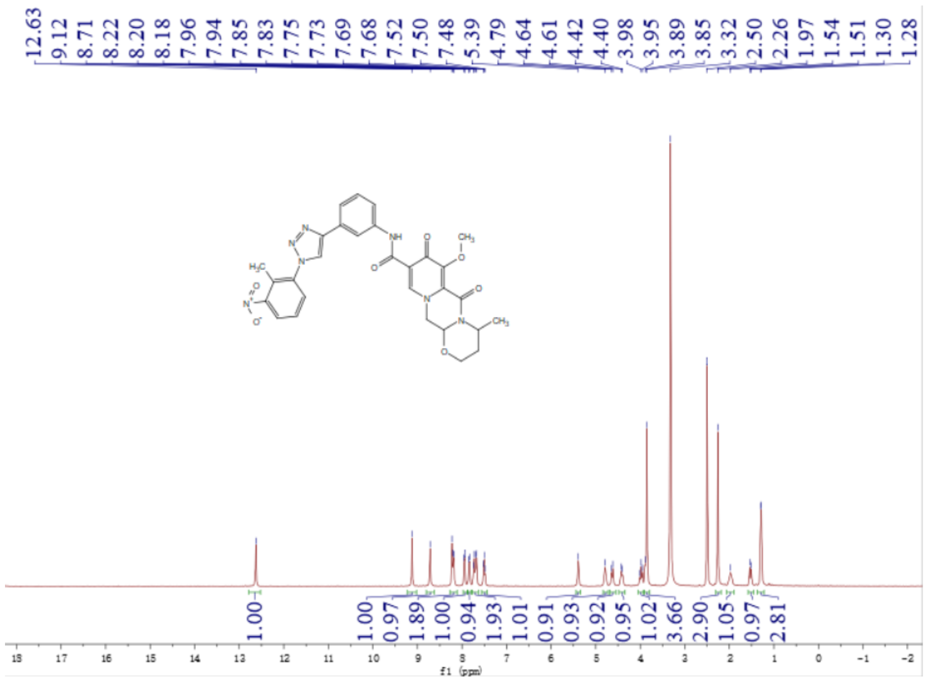

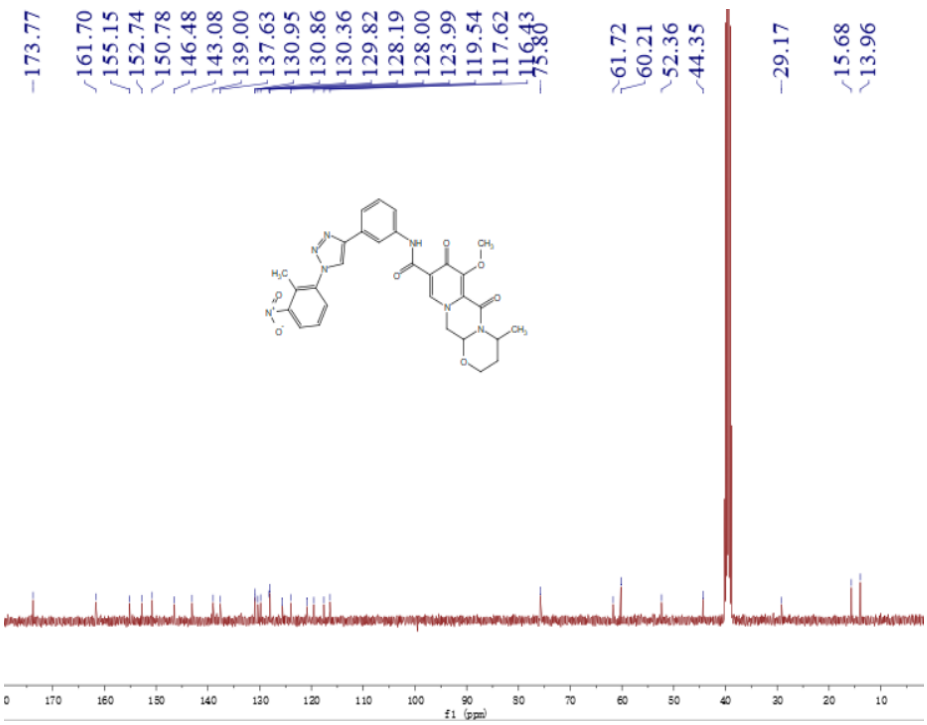

Figure S6. ^{1}H NMR and ^{13}C NMR spectrums of compound 5f

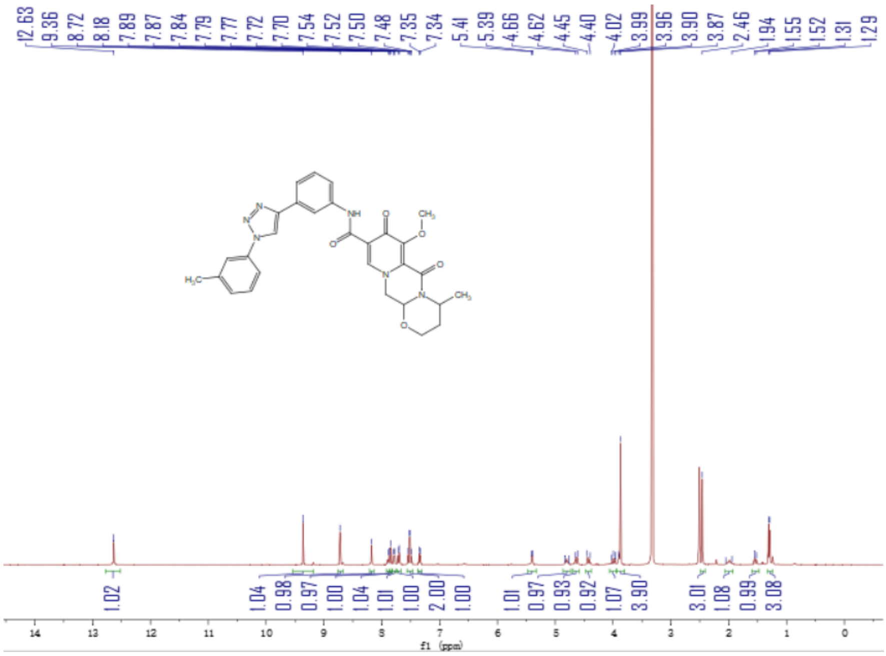

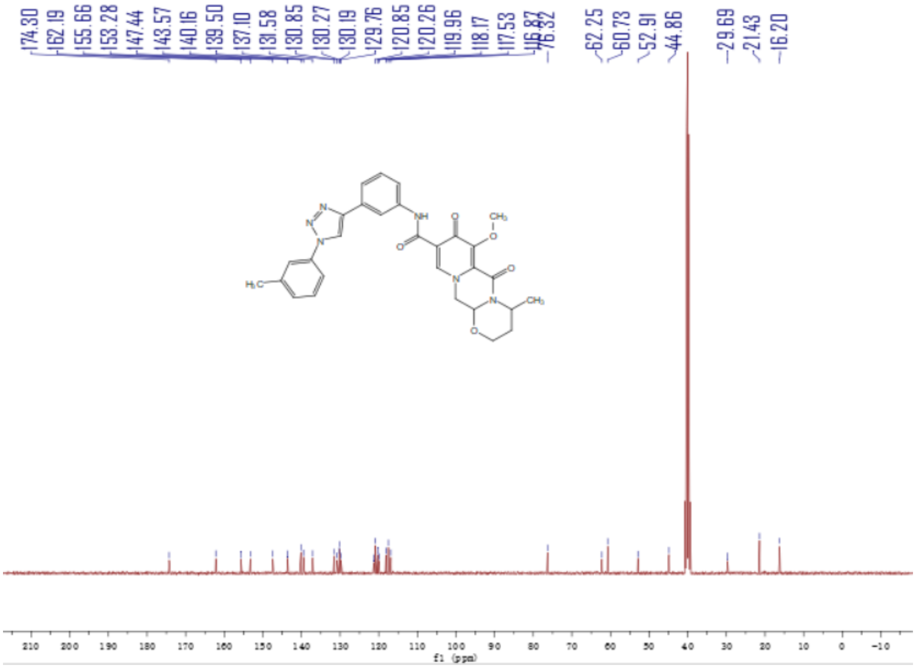

Figure S7. ^{1}H NMR and ^{13}C NMR spectrums of compound 5g

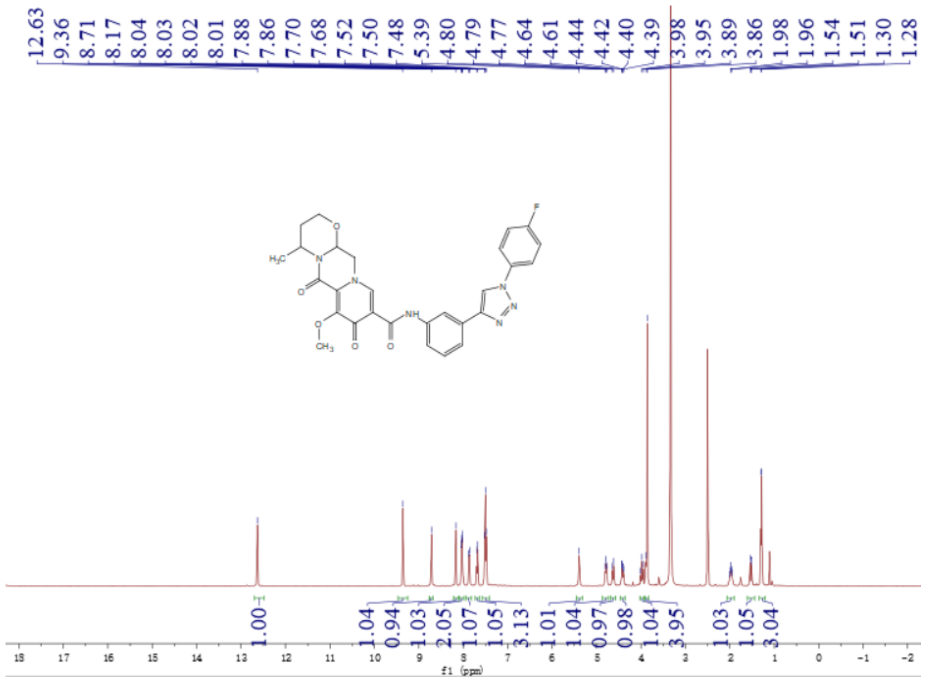

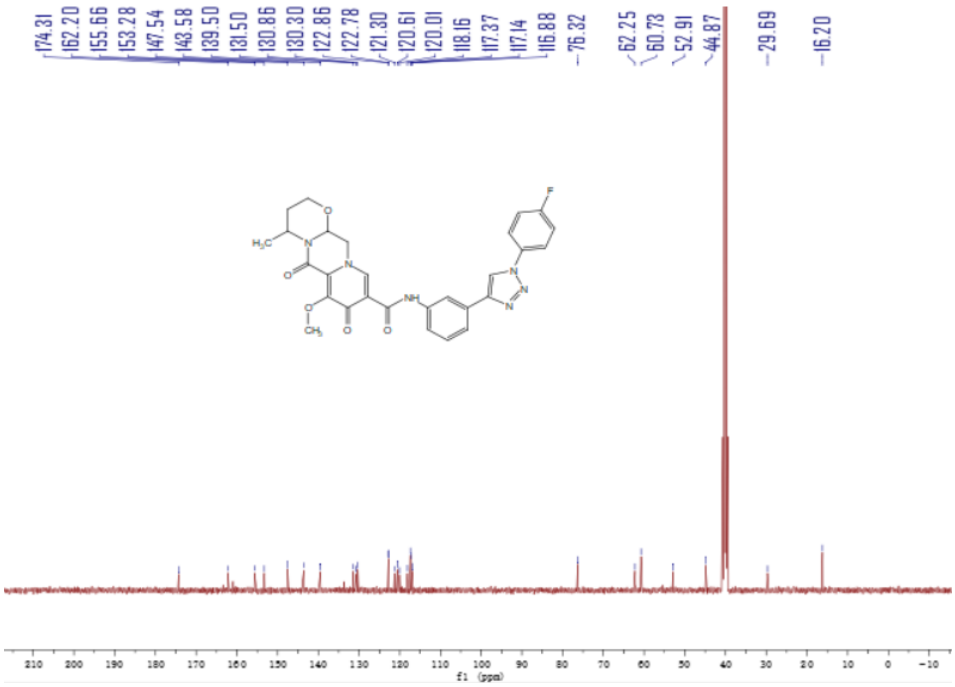

Figure S8. ^{1}H NMR and ^{13}C NMR spectrums of compound 5h

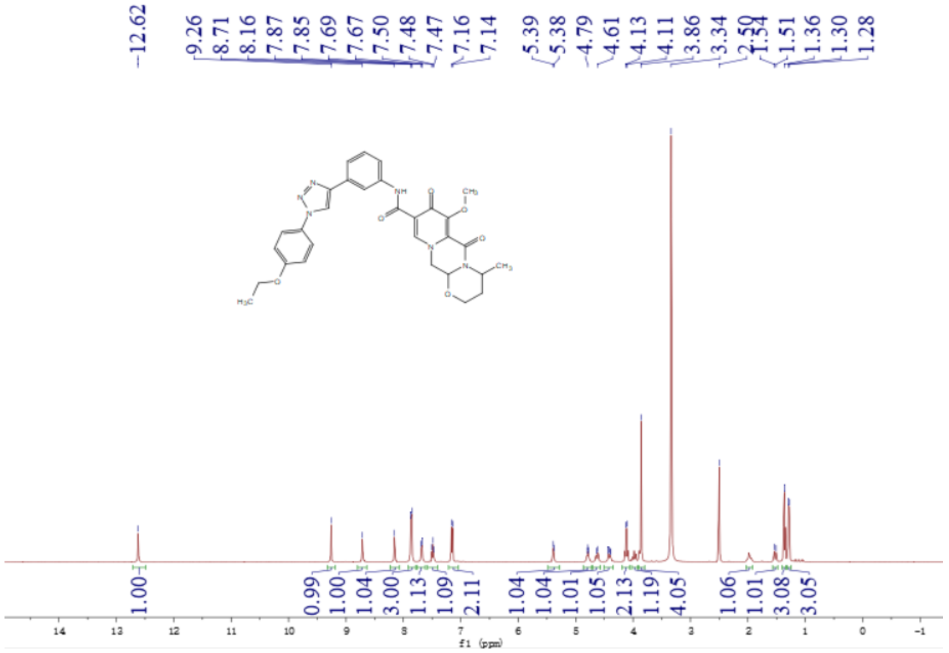

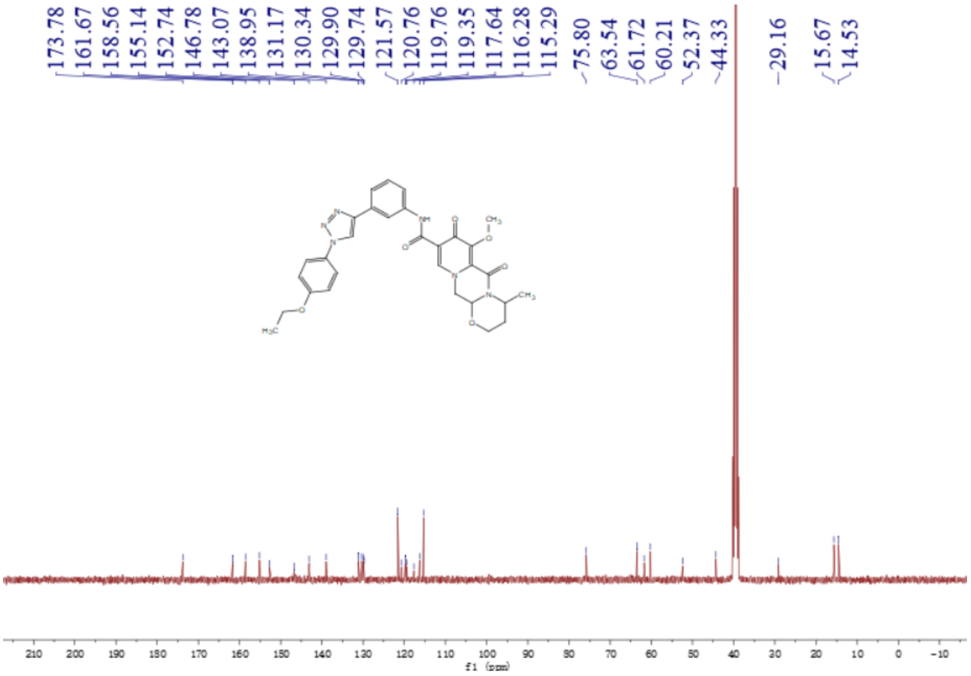

Figure S9. ^{1}H NMR and ^{13}C NMR spectrums of compound 5i

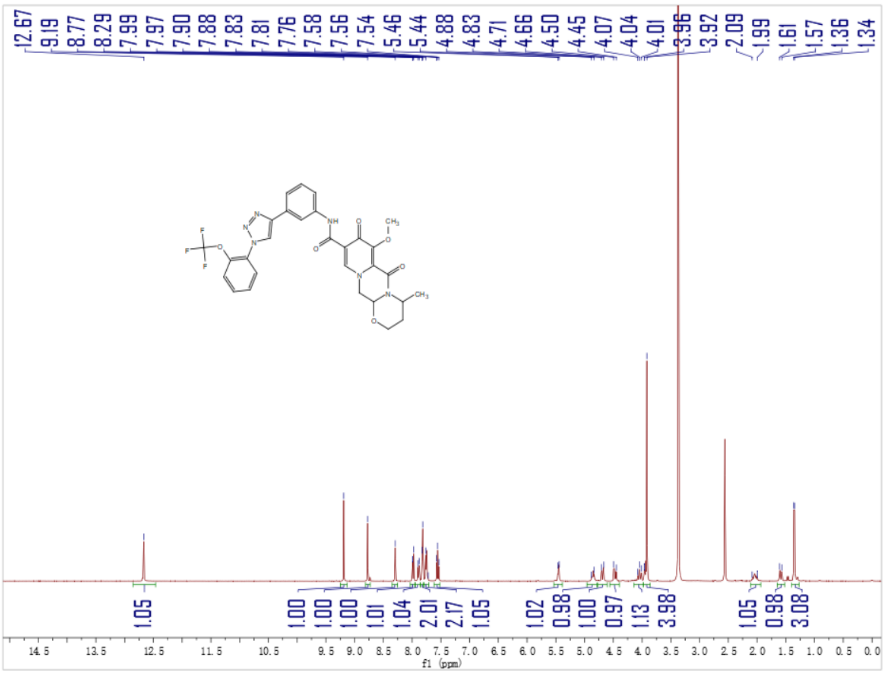

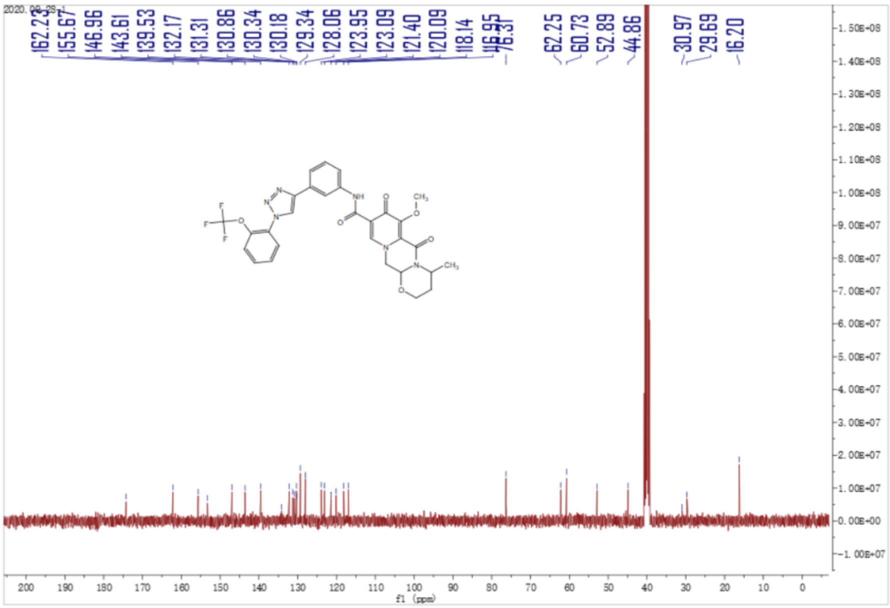

Figure S10. ^{1}H NMR and ^{13}C NMR spectrums of compound 5j

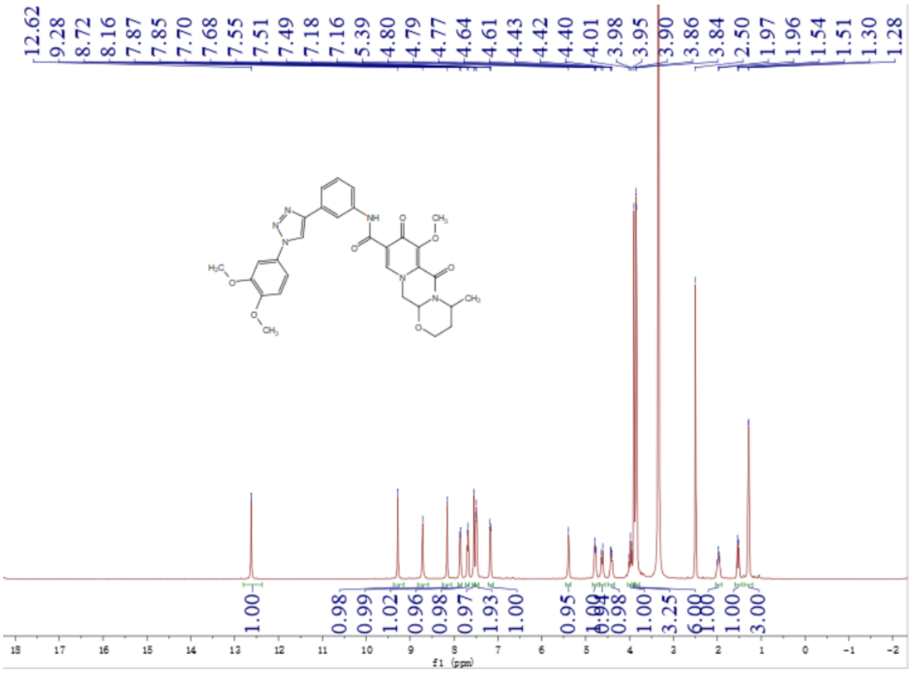

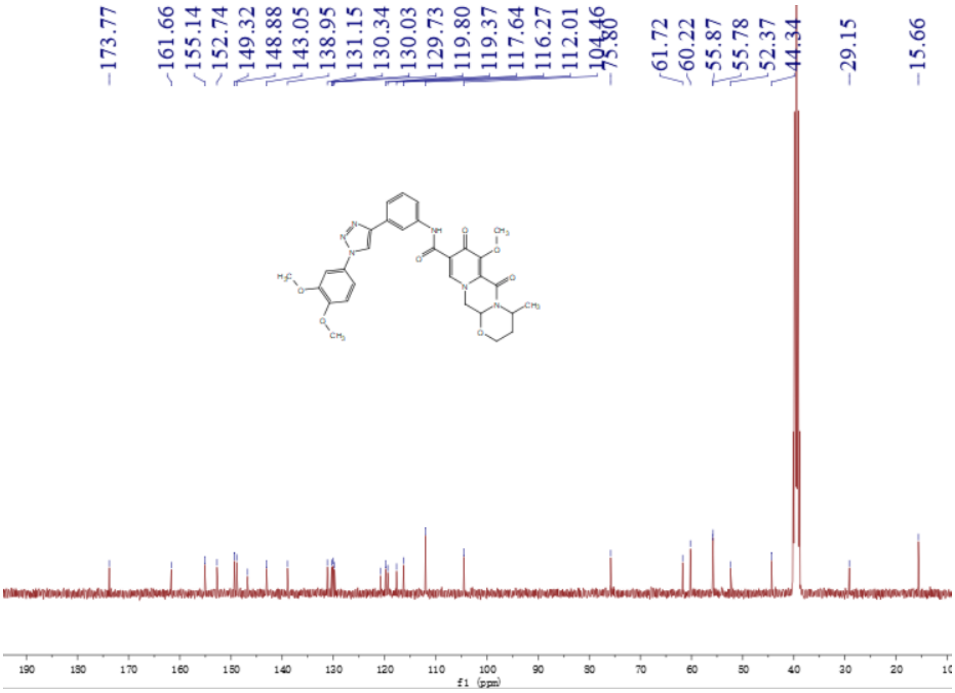

Figure S11. ^{1}H NMR and ^{13}C NMR spectrums of compound 5k

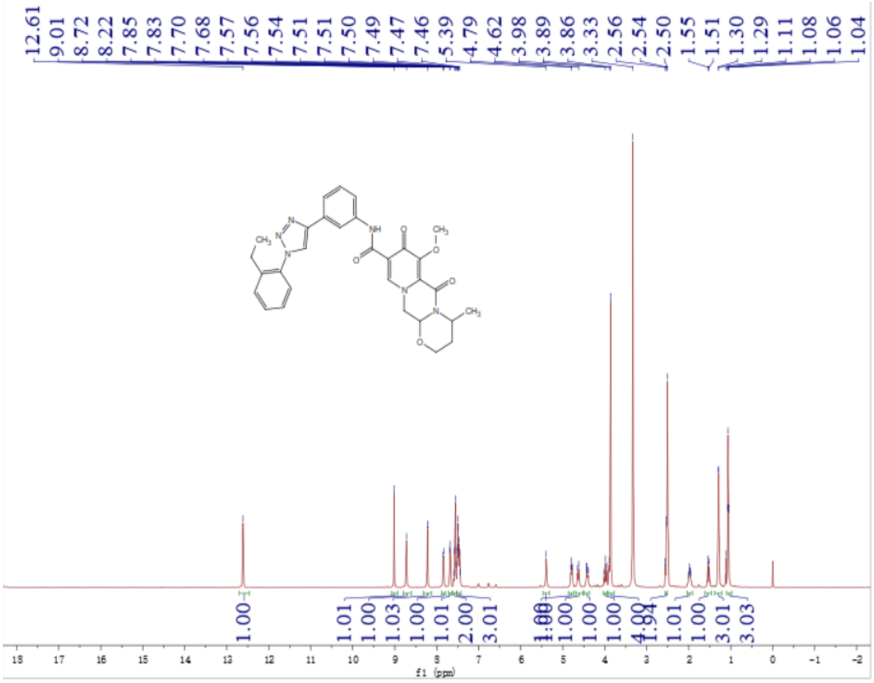

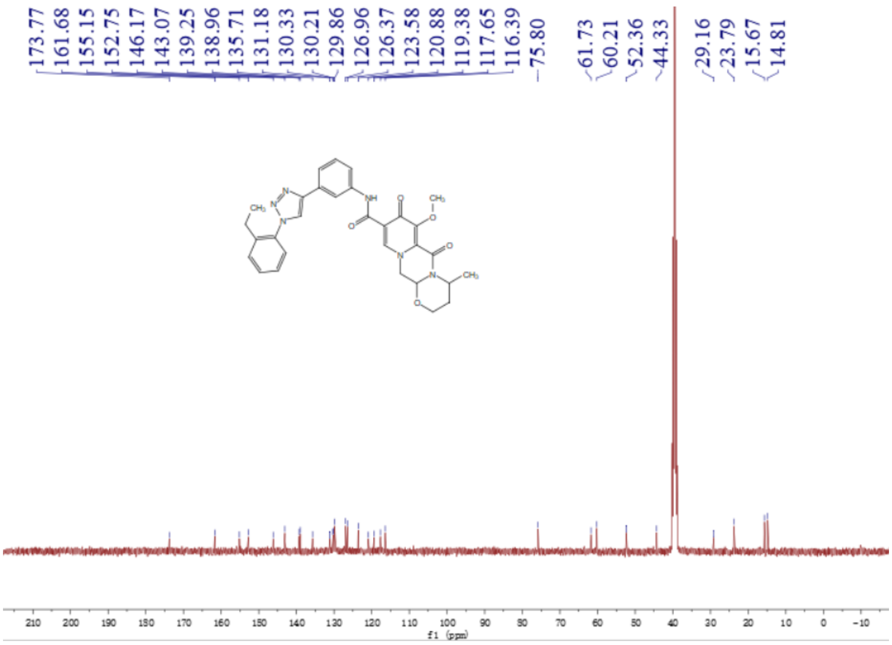

Figure S12. ^{1}H **NMR and** ^{13}C **NMR spectrums of compound 5l**

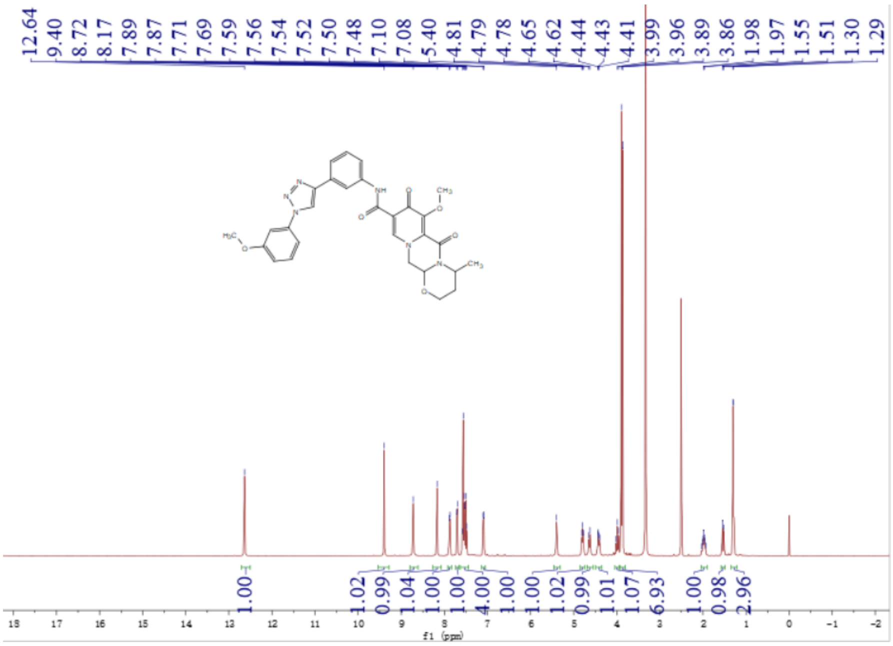

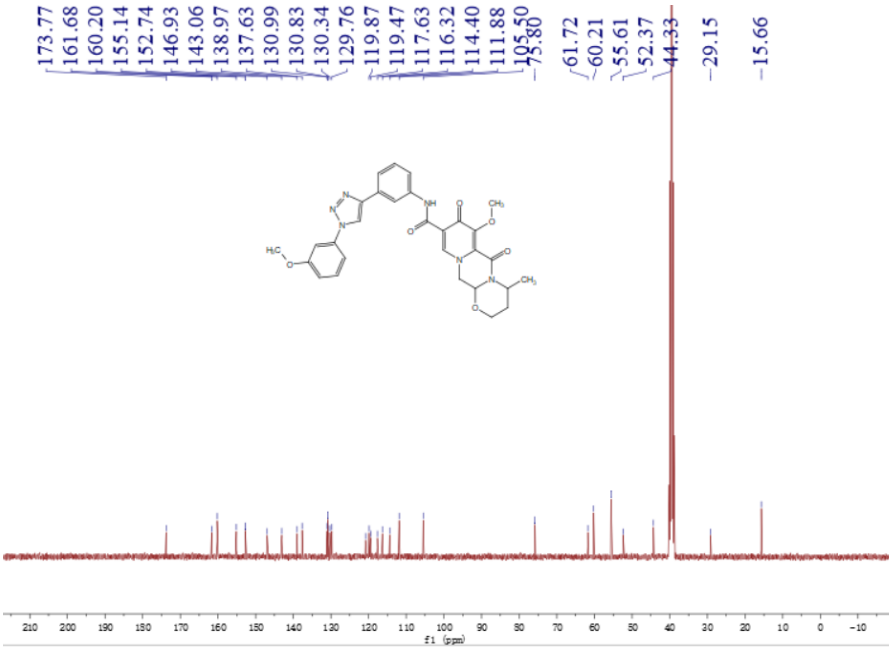

Figure S13. ^{1}H NMR and ^{13}C NMR spectrums of compound 5m

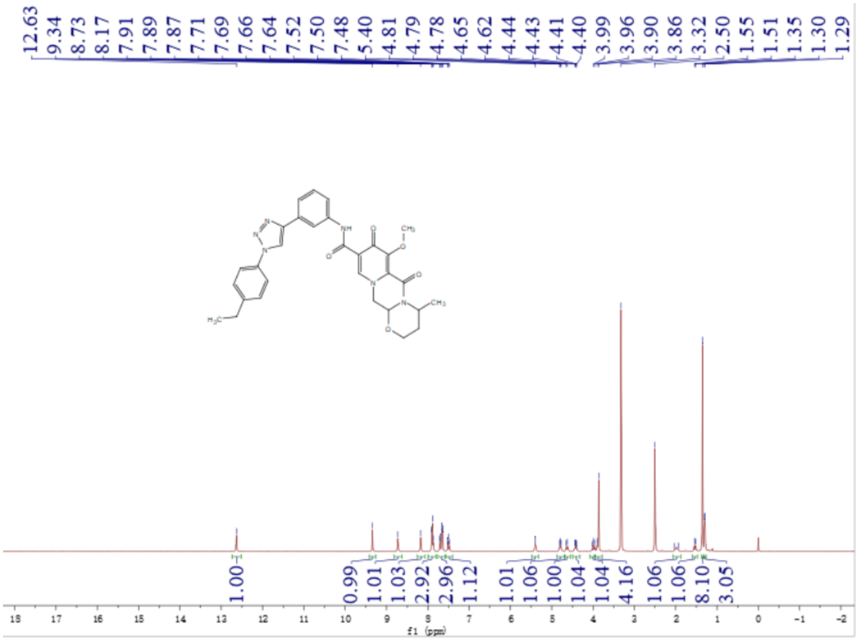

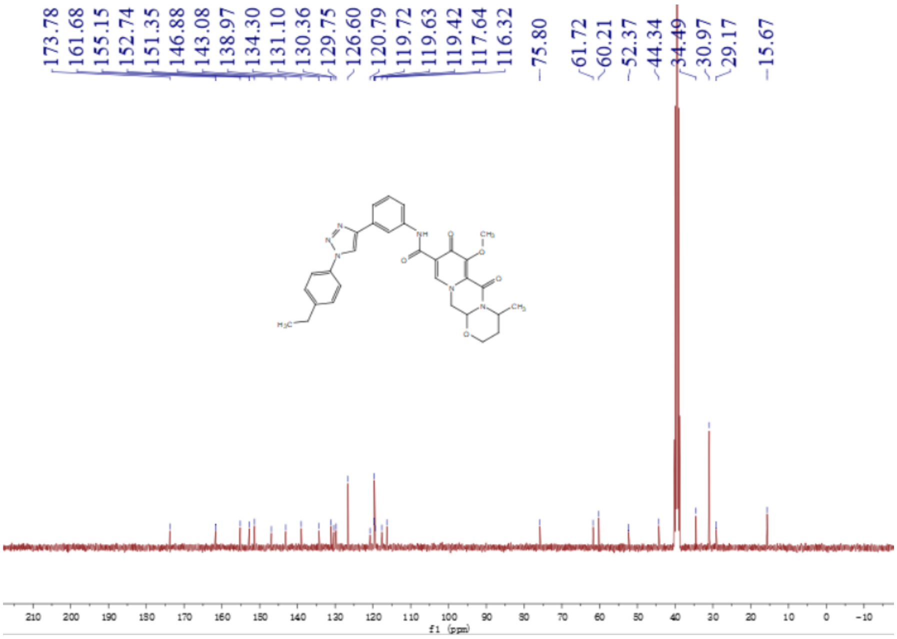

Figure S14. ^{1}H NMR and ^{13}C NMR spectrums of compound 5n

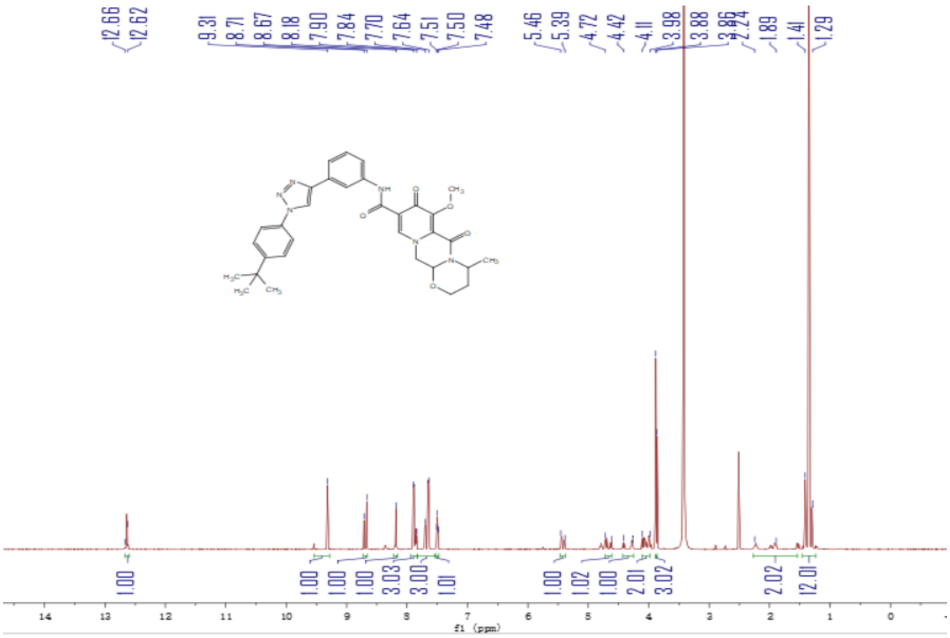

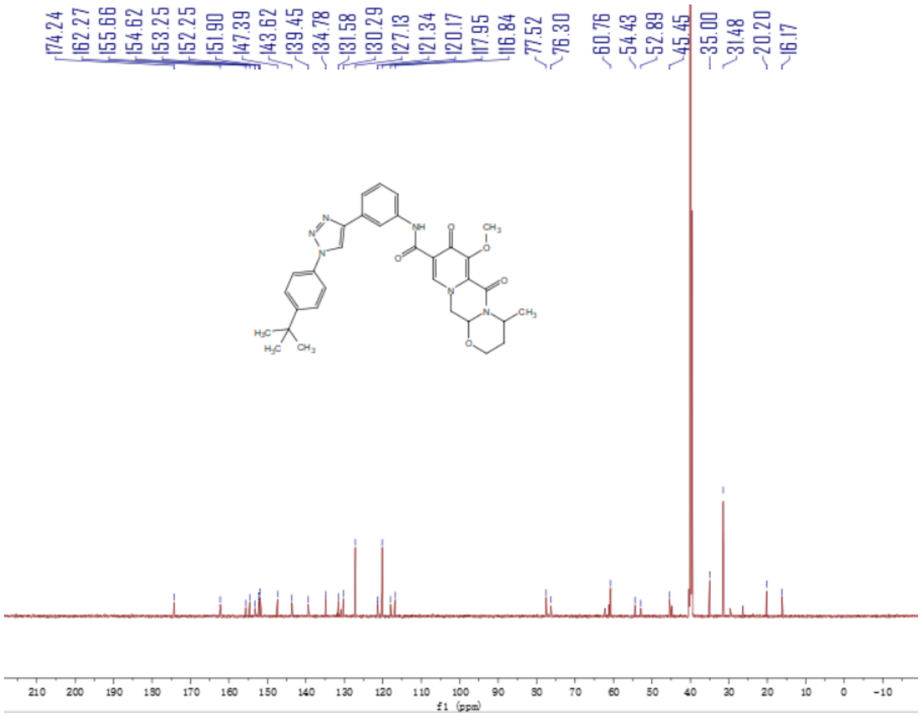

Figure S15. ^{1}H NMR and ^{13}C NMR spectrums of compound 5o

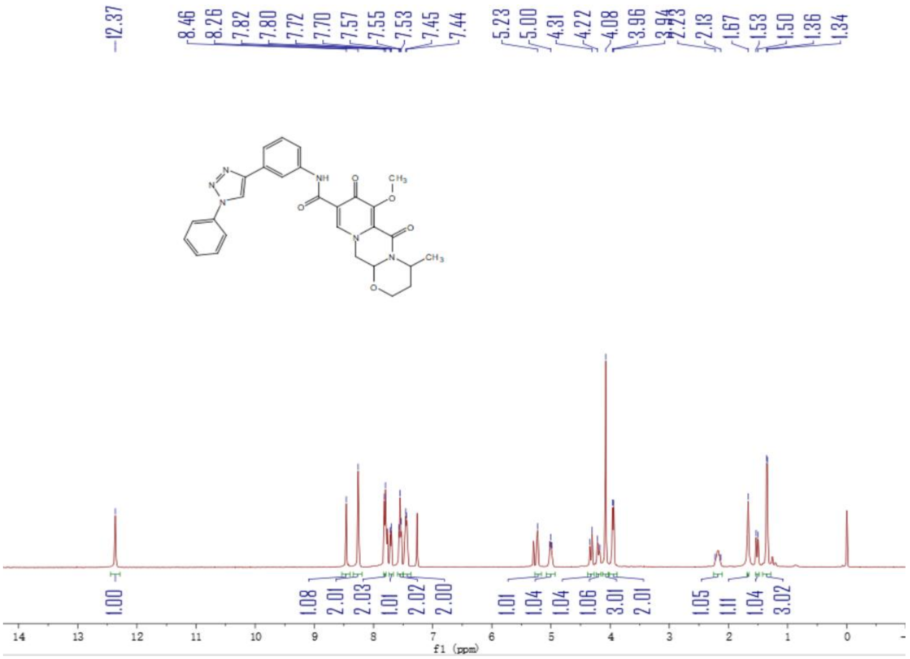

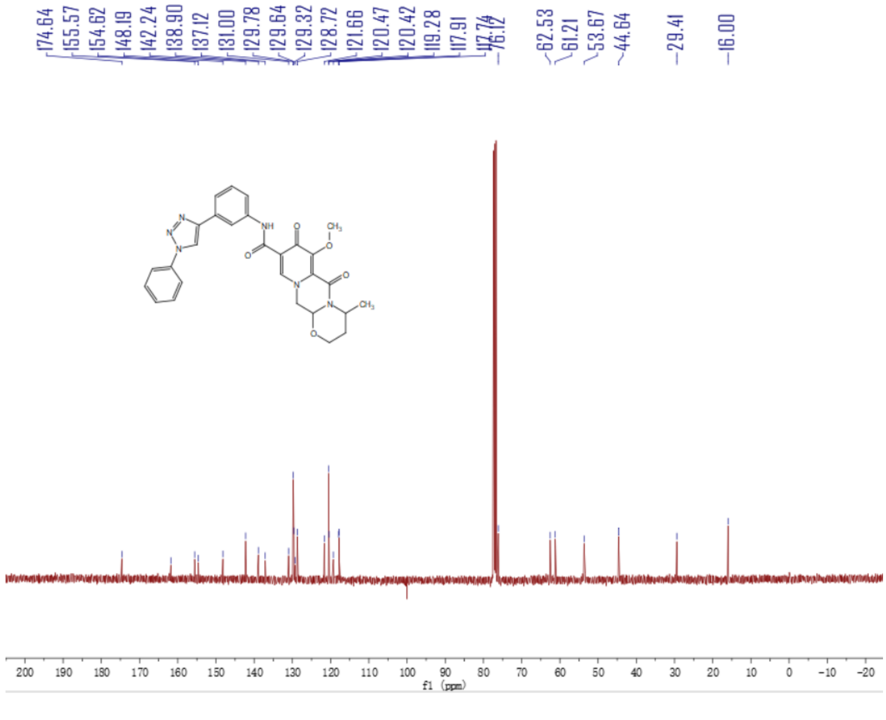

Figure S16. ^{1}H NMR and ^{13}C NMR spectrums of compound 5p

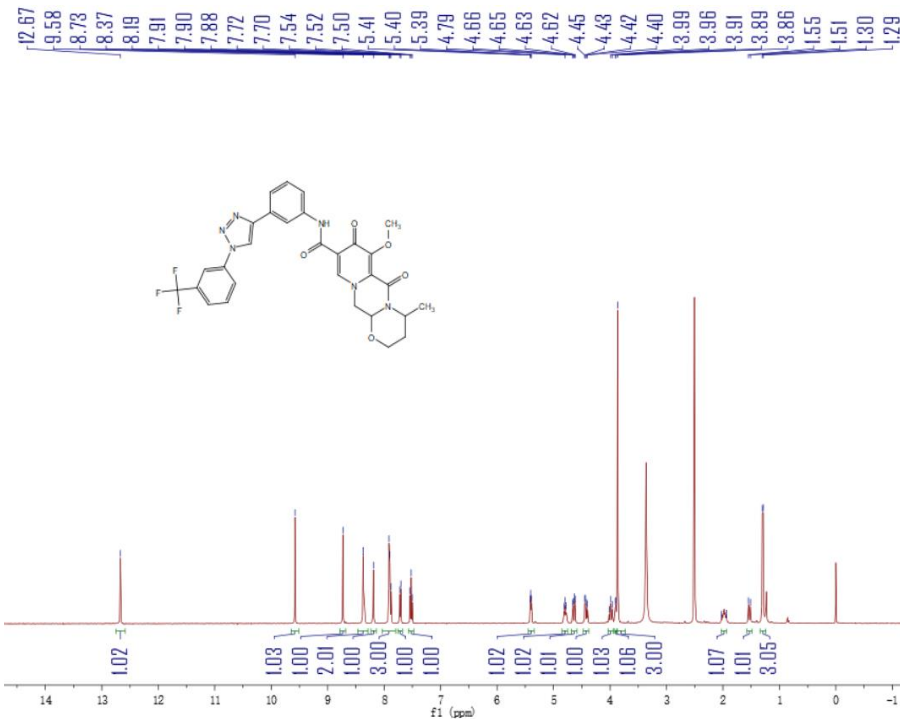

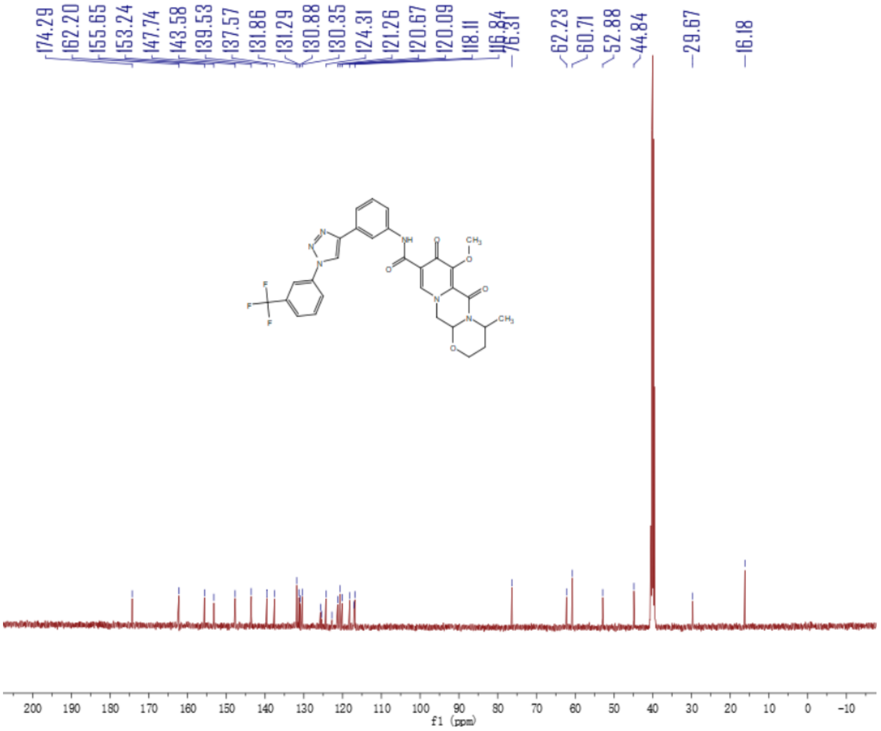

Figure S17. ^{1}H NMR and ^{13}C NMR spectrums of compound 5q

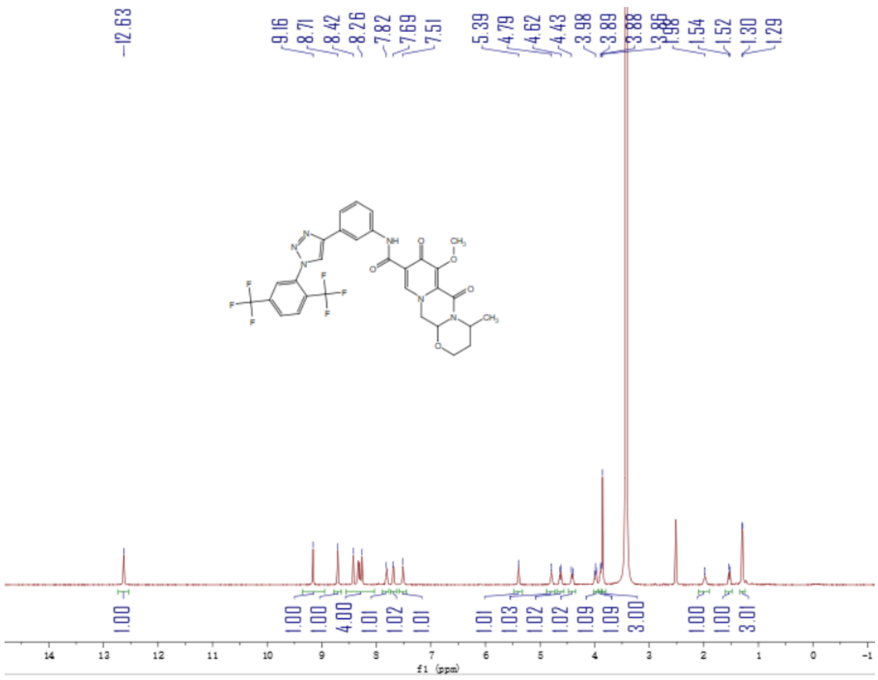

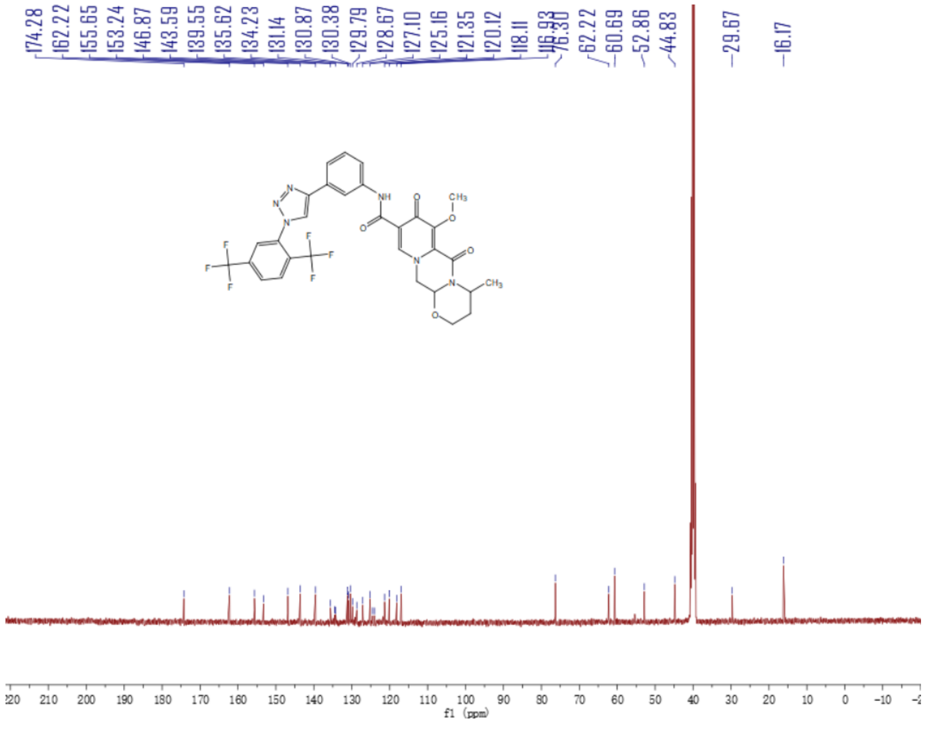

Figure S18. ^{1}H NMR and ^{13}C NMR spectrums of compound 5r

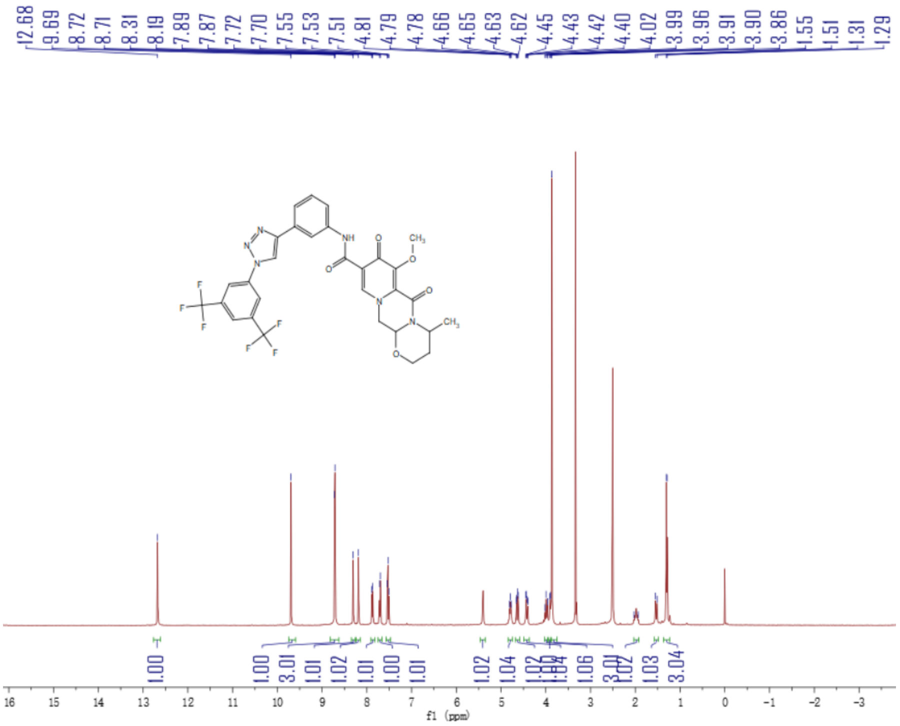

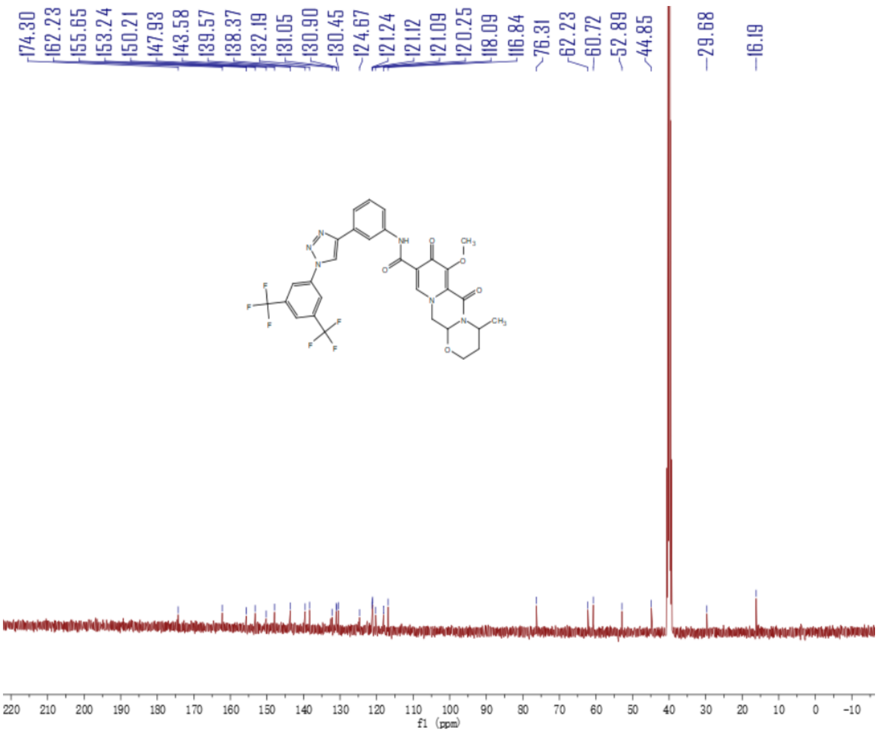

Figure S19. ^{1}H NMR and ^{13}C NMR spectrums of compound 5s

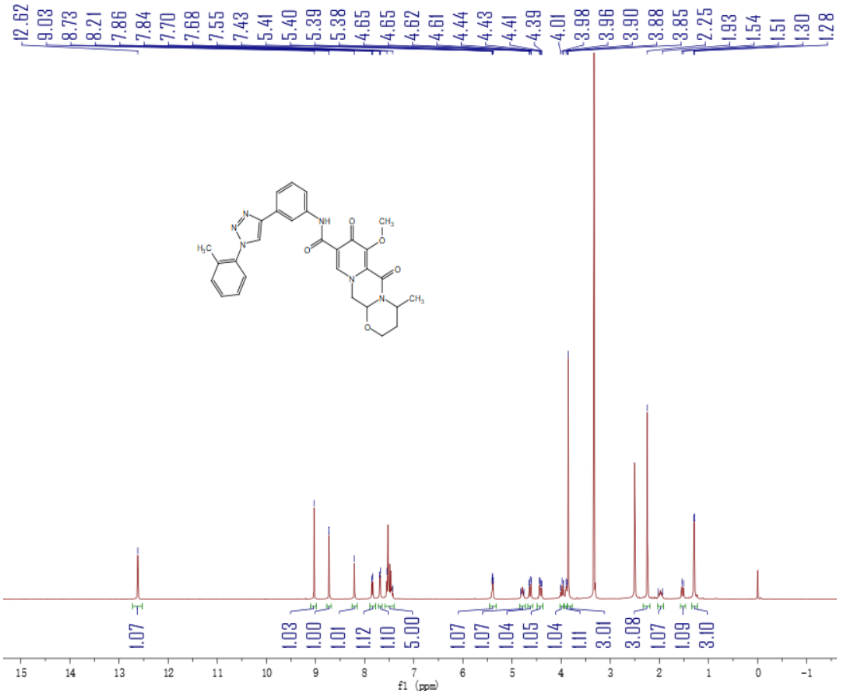

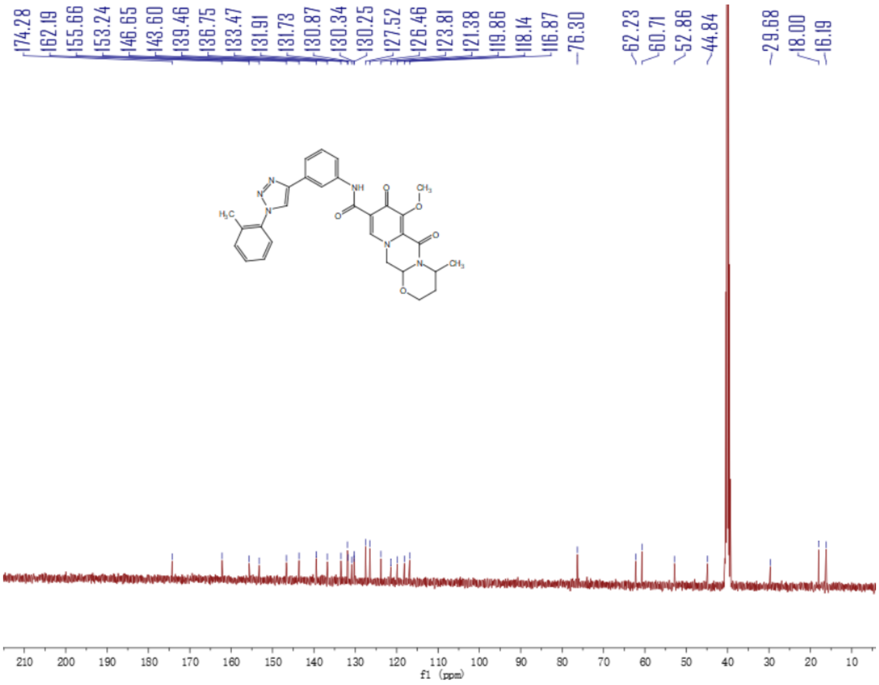

Figure S20. ^{1}H NMR and ^{13}C NMR spectrums of compound 5t

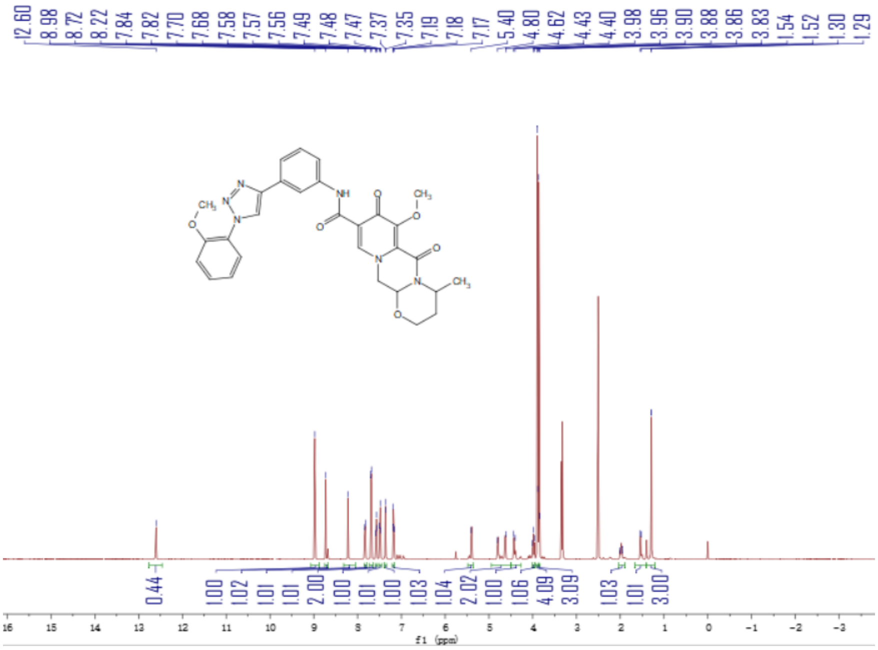

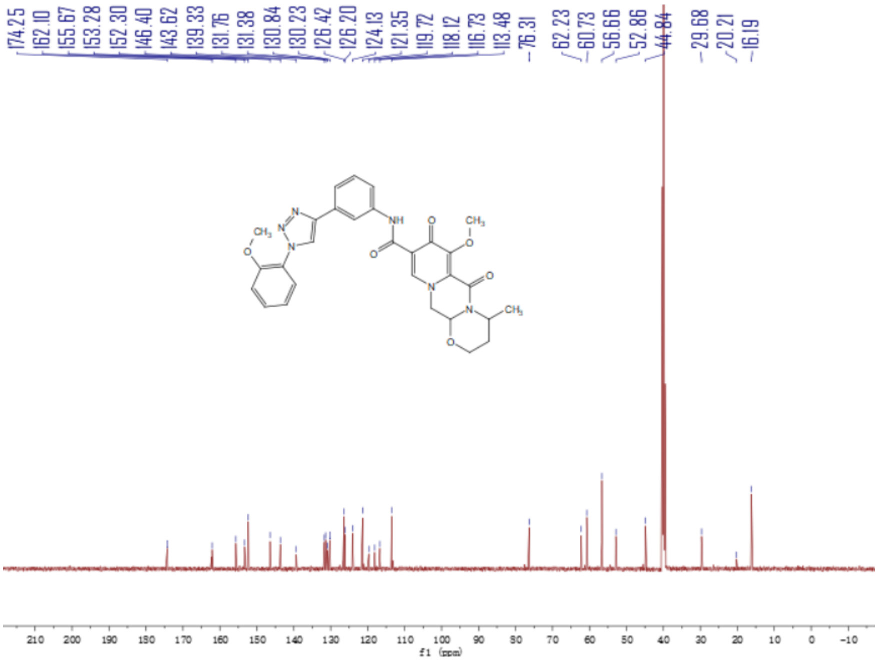

Figure S21. ^{1}H NMR and ^{13}C NMR spectrums of compound 5u

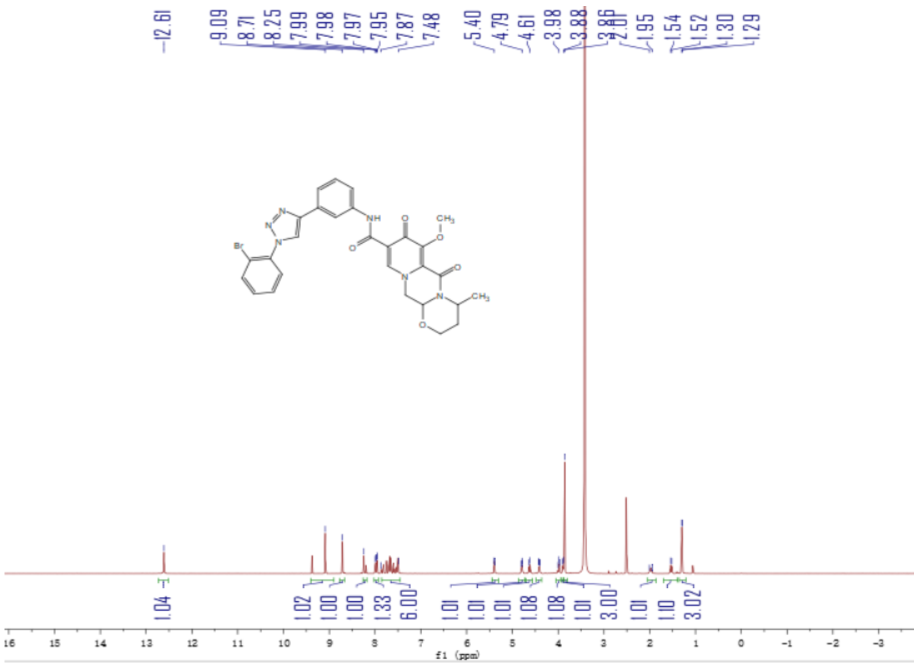

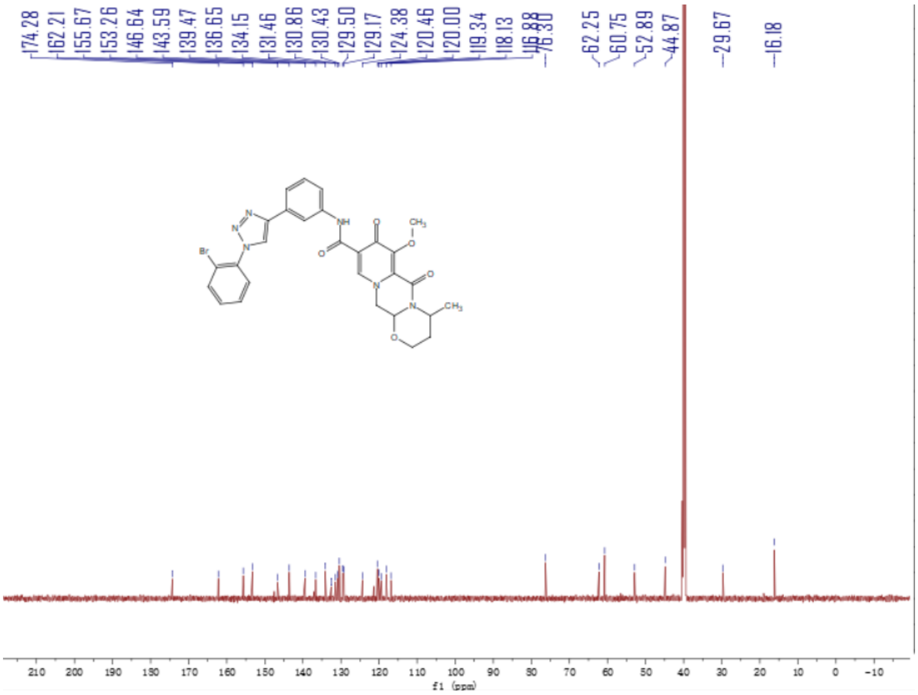

Figure S22. ^{1}H NMR and ^{13}C NMR spectrums of compound 5v

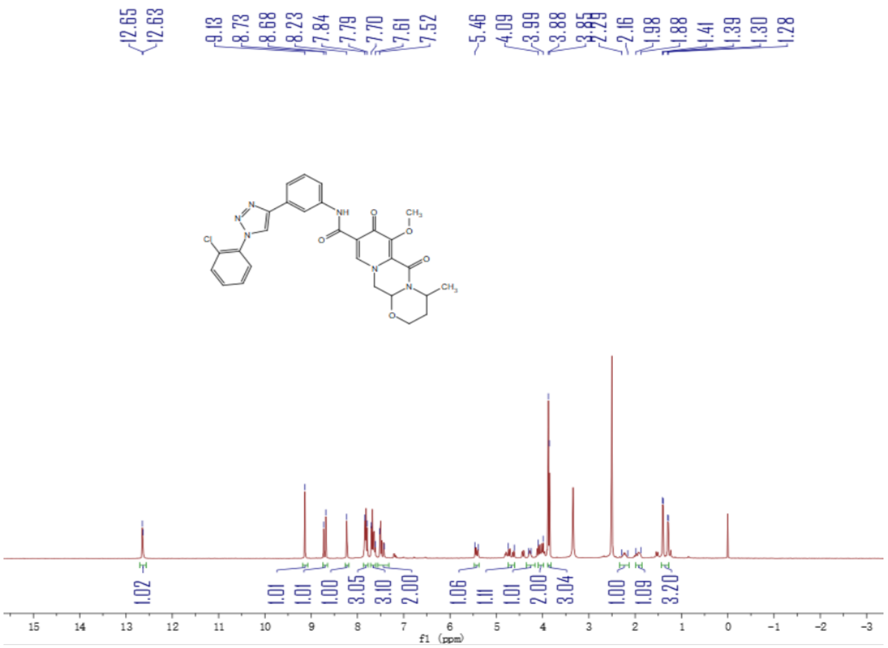

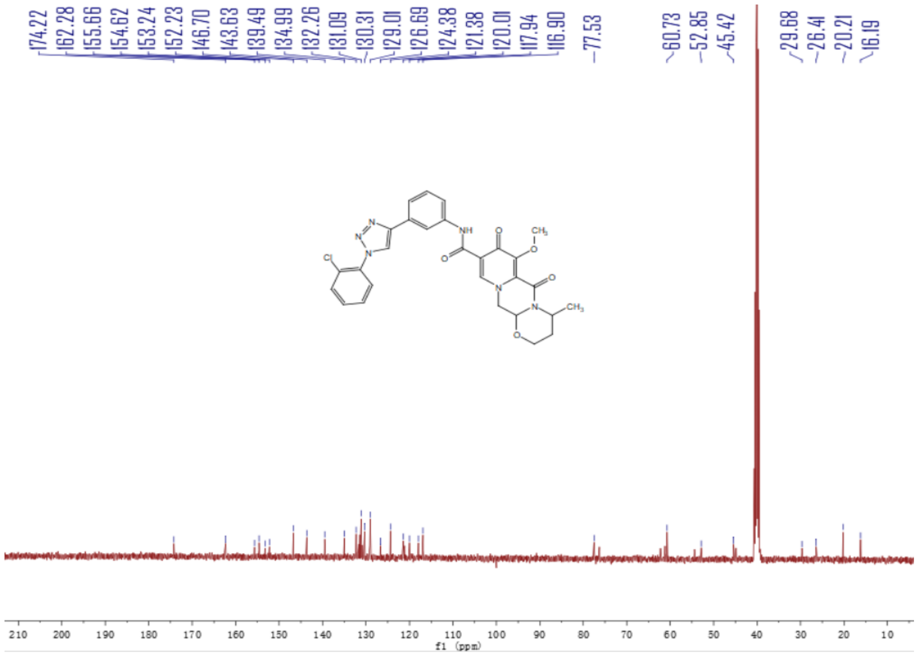

Figure S23. ^{1}H NMR and ^{13}C NMR spectrums of compound 5w

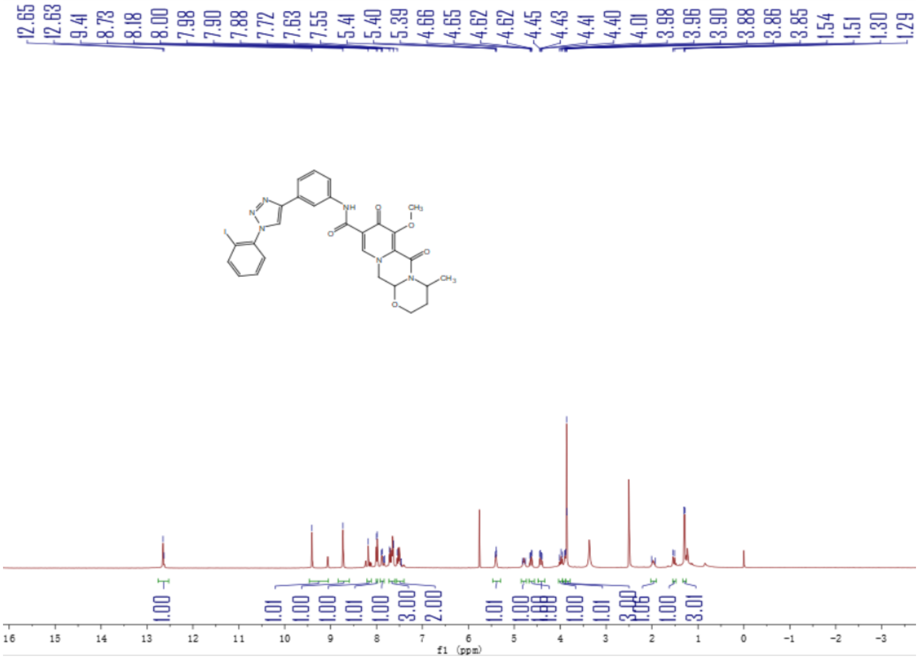

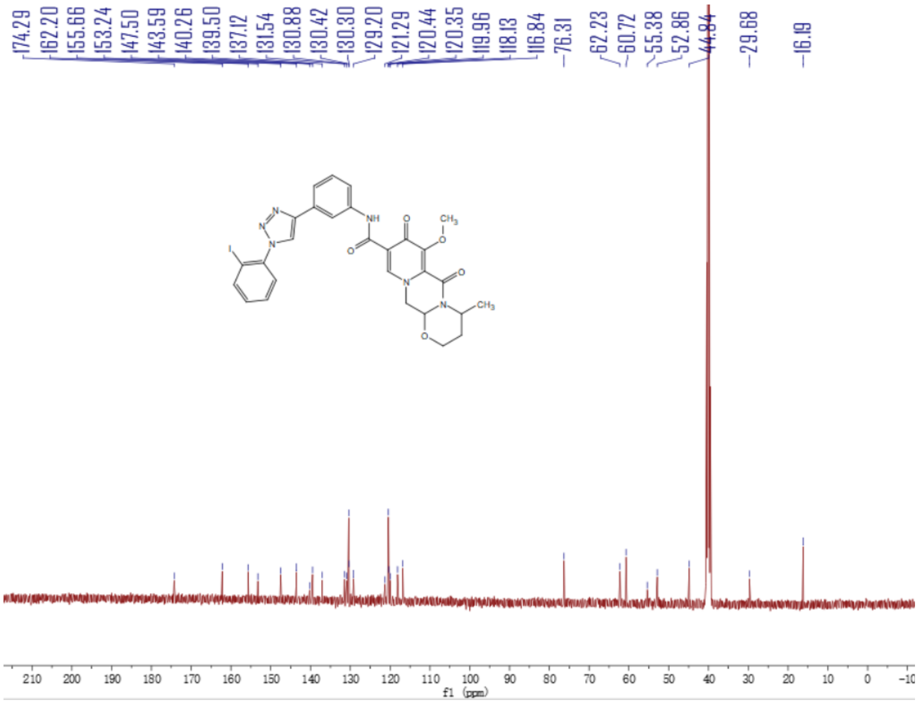

Figure S24. ¹H NMR and ¹³C NMR spectrums of compound 5x

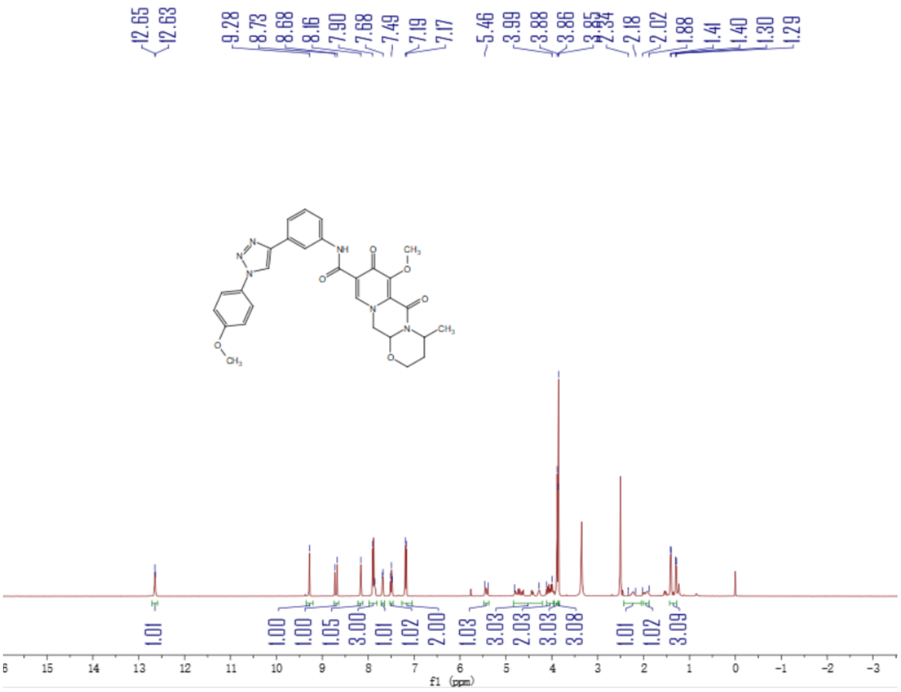

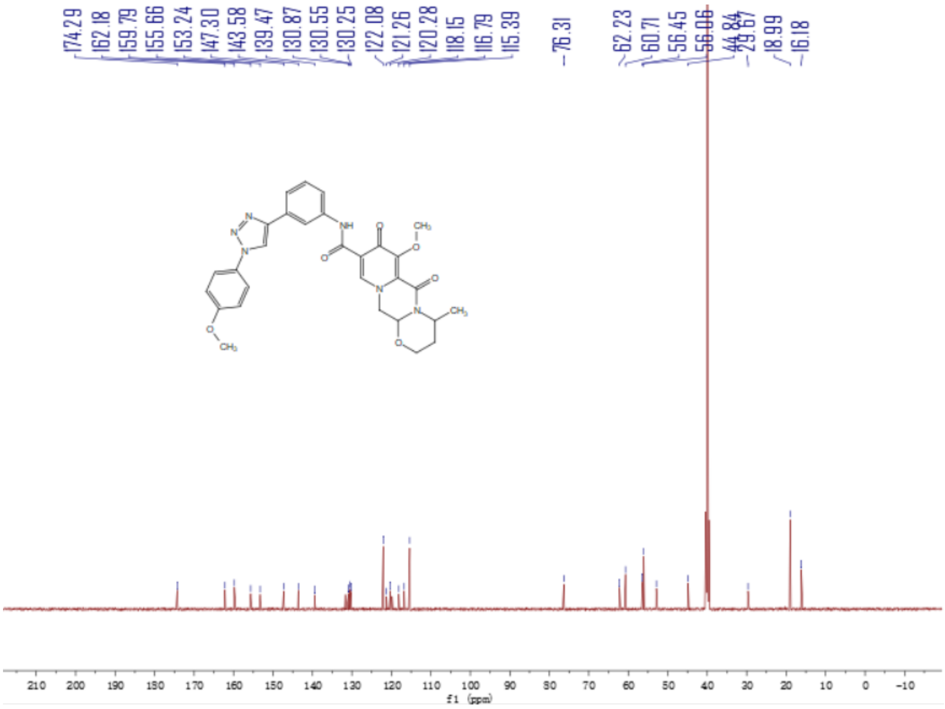

Figure S25. ^{1}H NMR and ^{13}C NMR spectrums of compound 5y

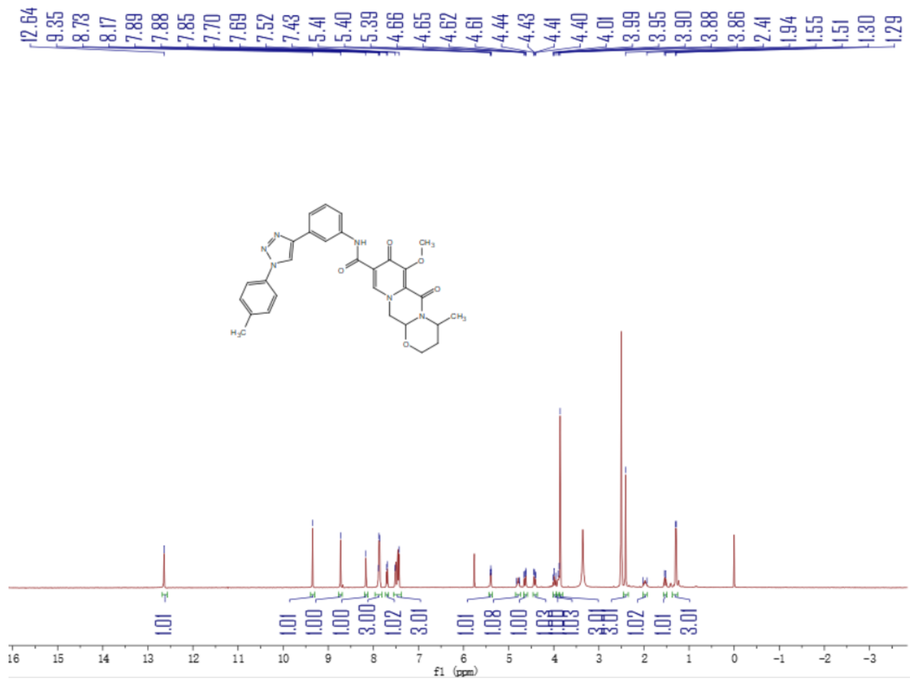

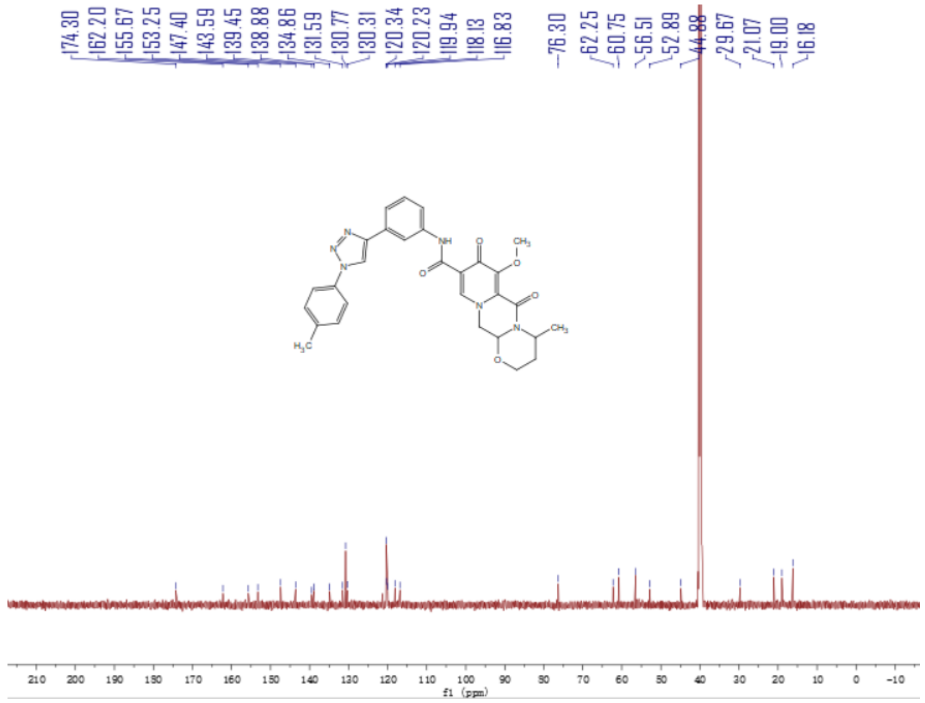

Figure S26. ^{1}H NMR and ^{13}C NMR spectrums of compound 5z

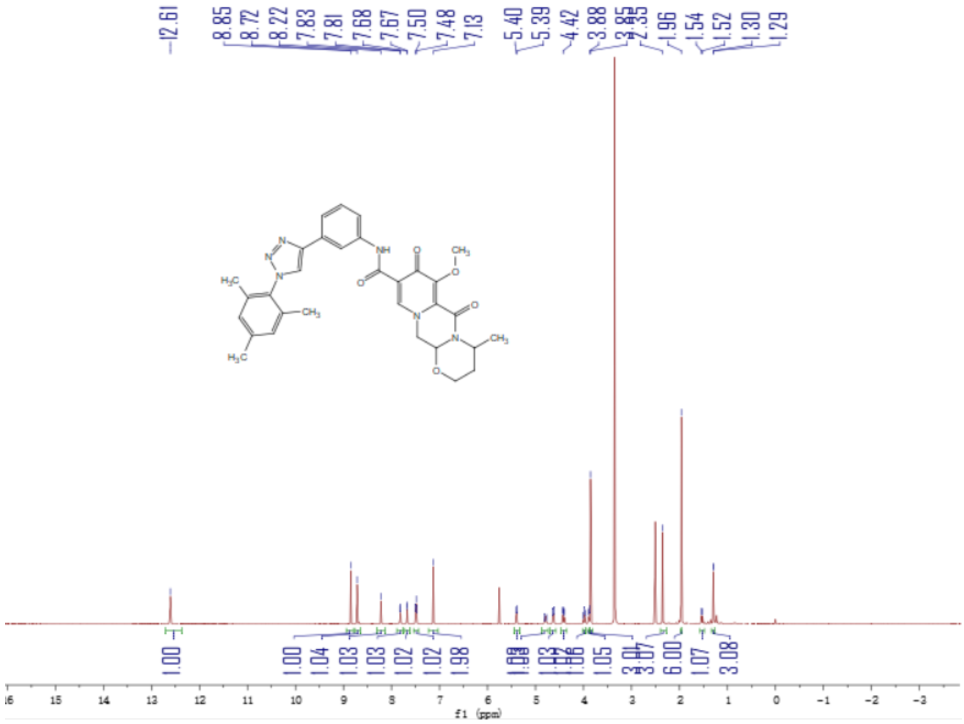

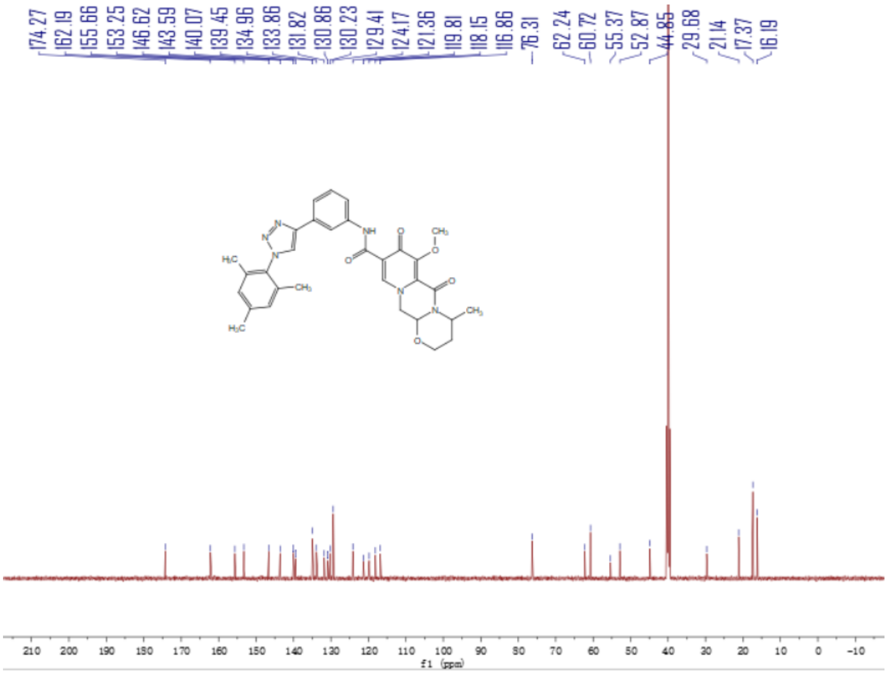

Figure S27. ^{1}H NMR and ^{13}C NMR spectrums of compound 6a

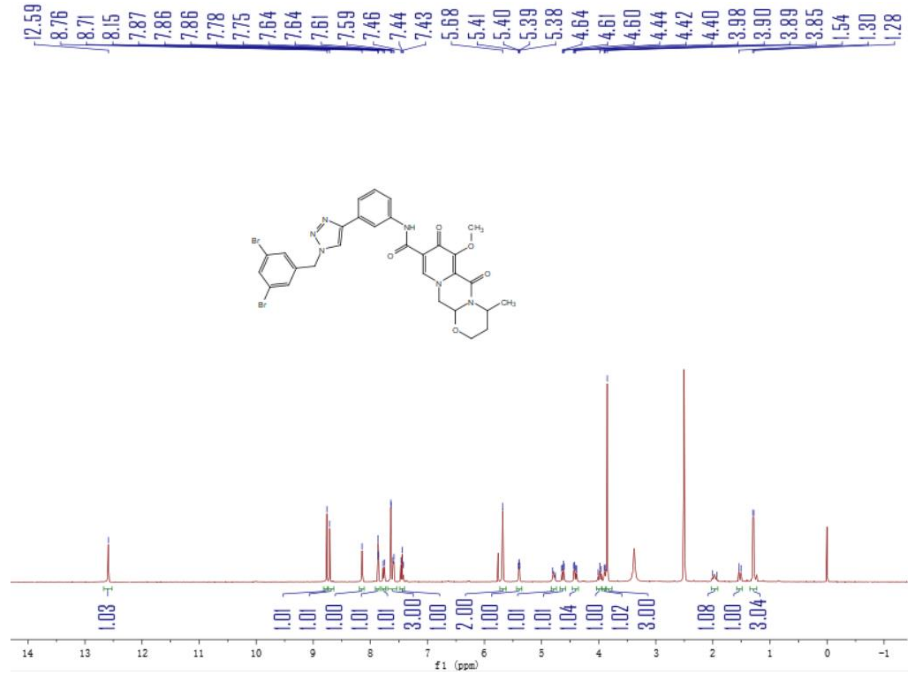

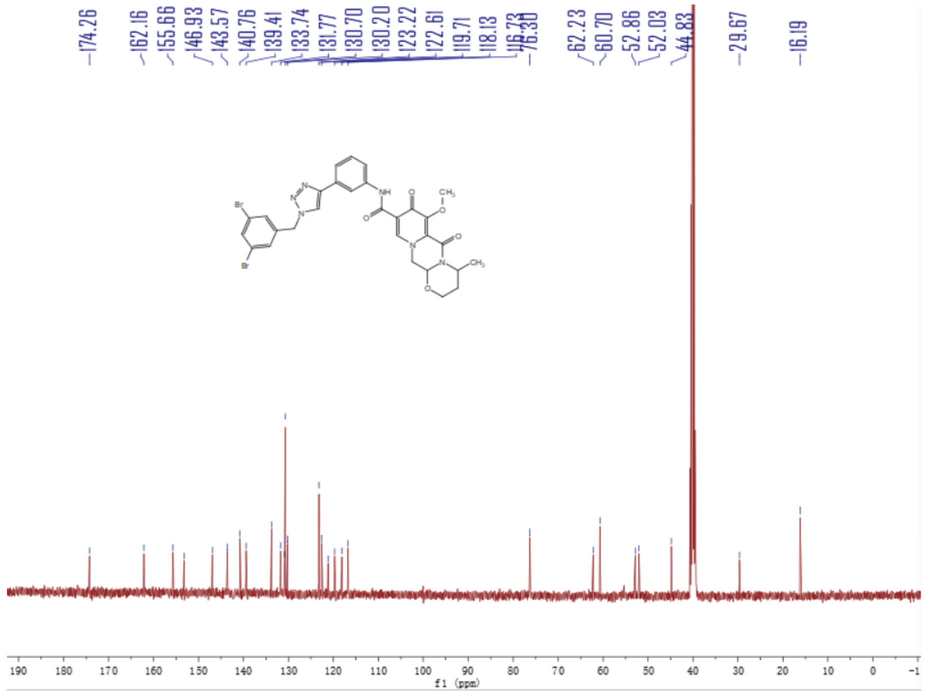

Figure S28. ^{1}H NMR and ^{13}C NMR spectrums of compound 6b

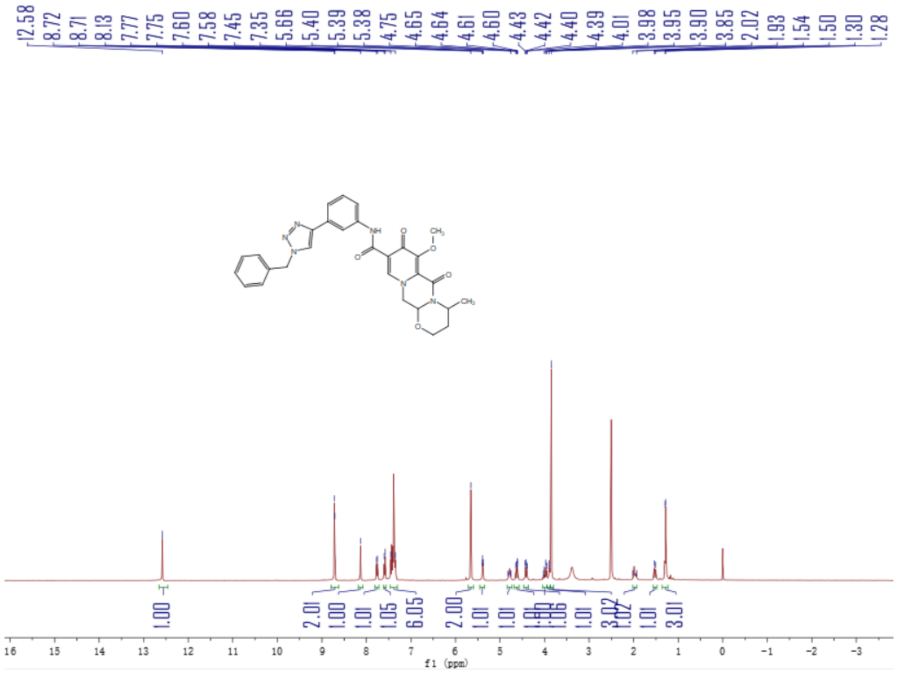

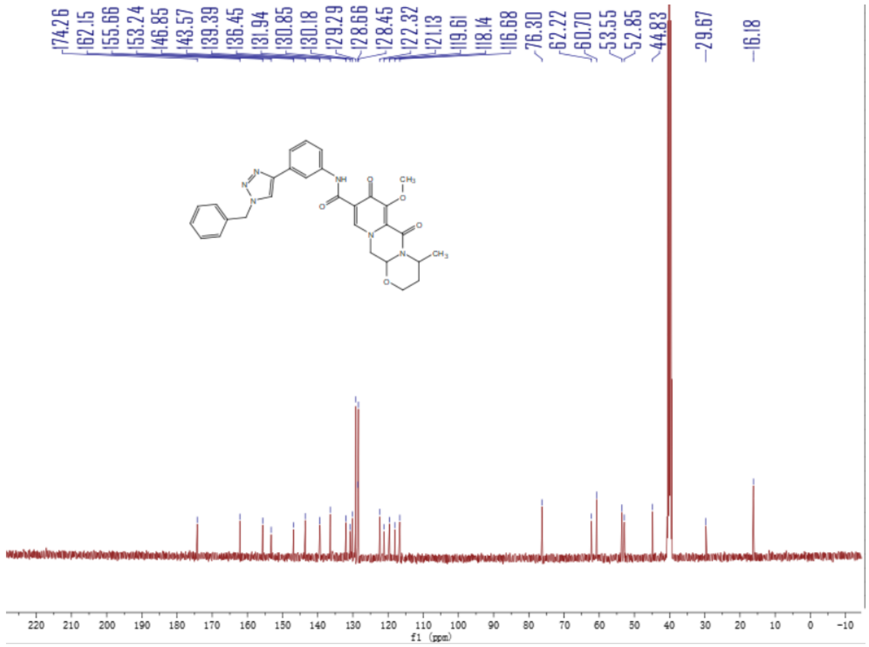

Figure S29. ^{1}H **NMR and** ^{13}C **NMR spectrums of compound 6c**

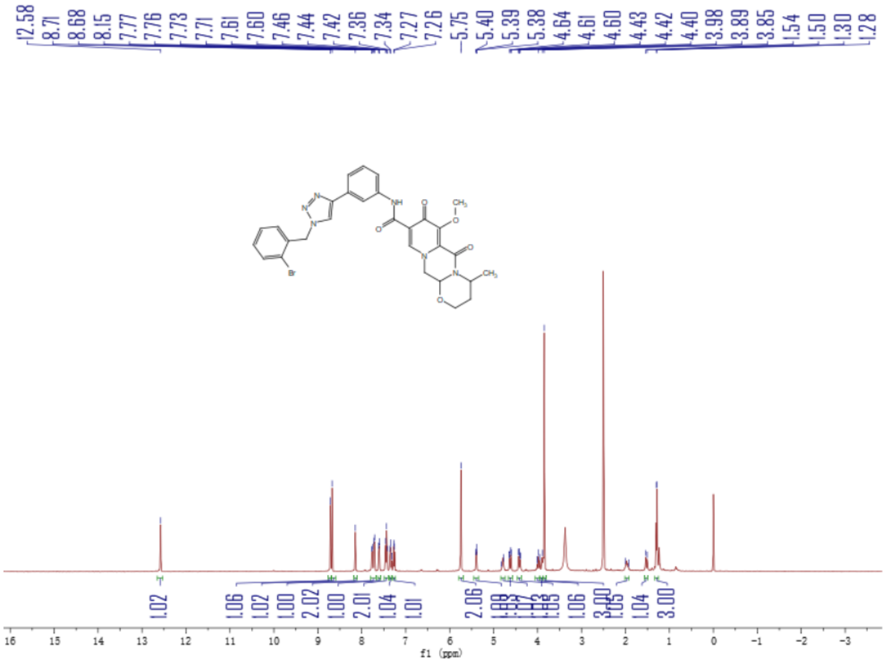

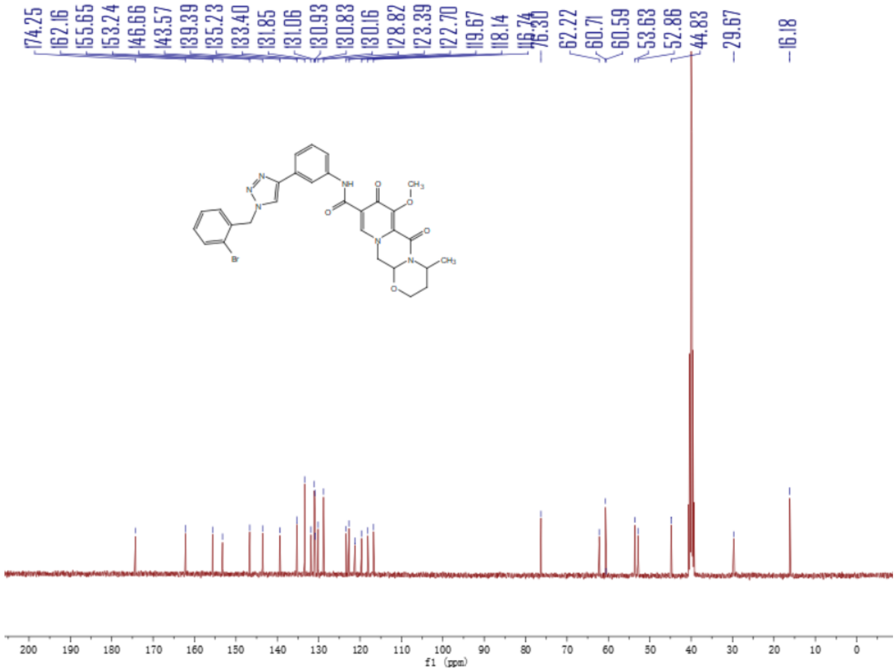

Figure S30. ^{1}H NMR and ^{13}C NMR spectrums of compound 6d

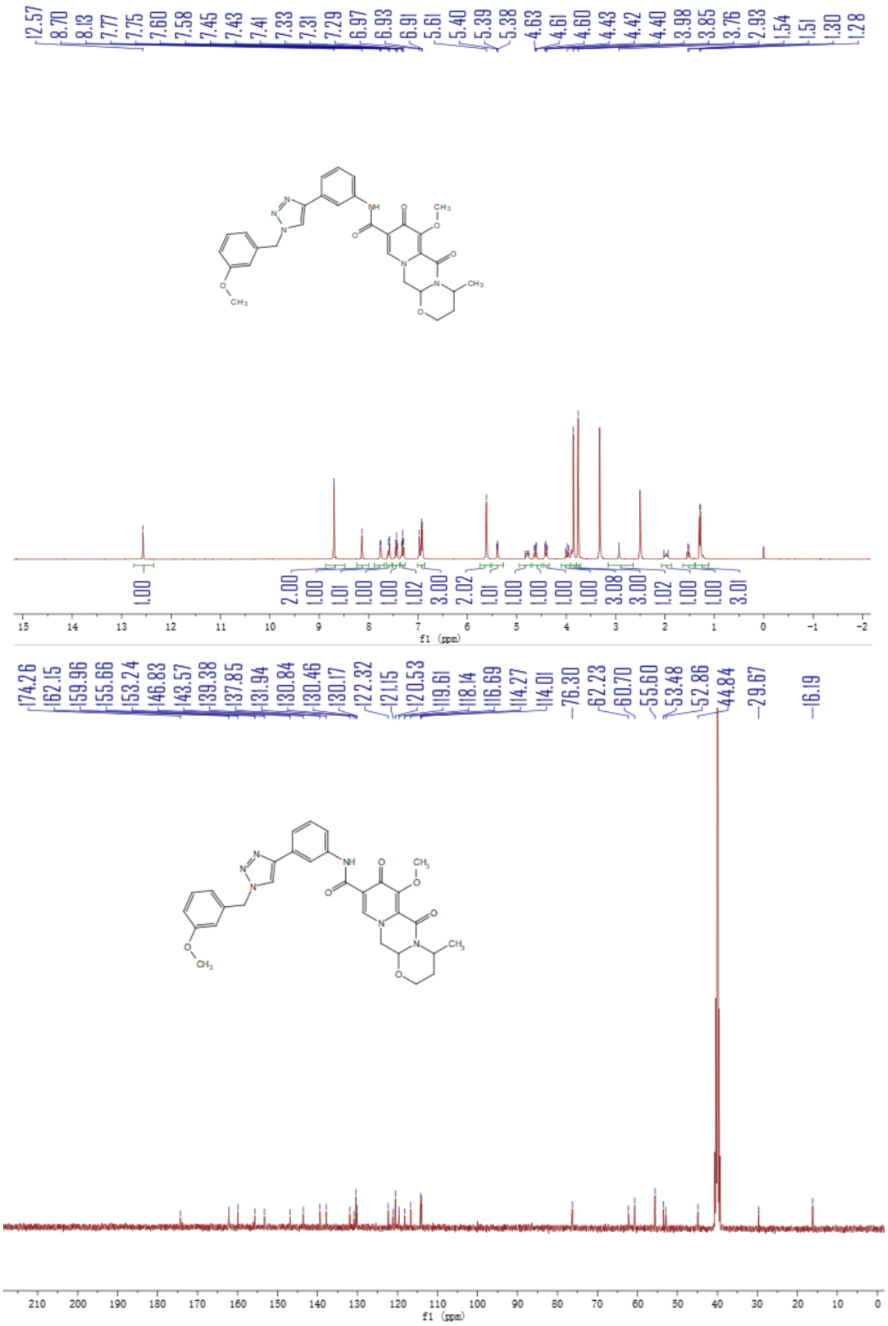

Figure S31. ^{1}H NMR and ^{13}C NMR spectrums of compound 6e

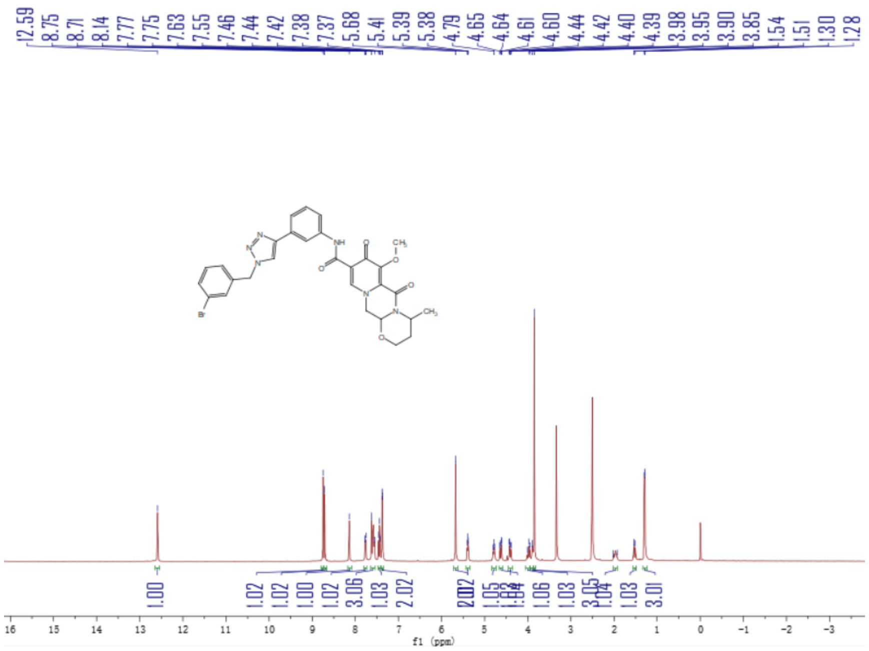

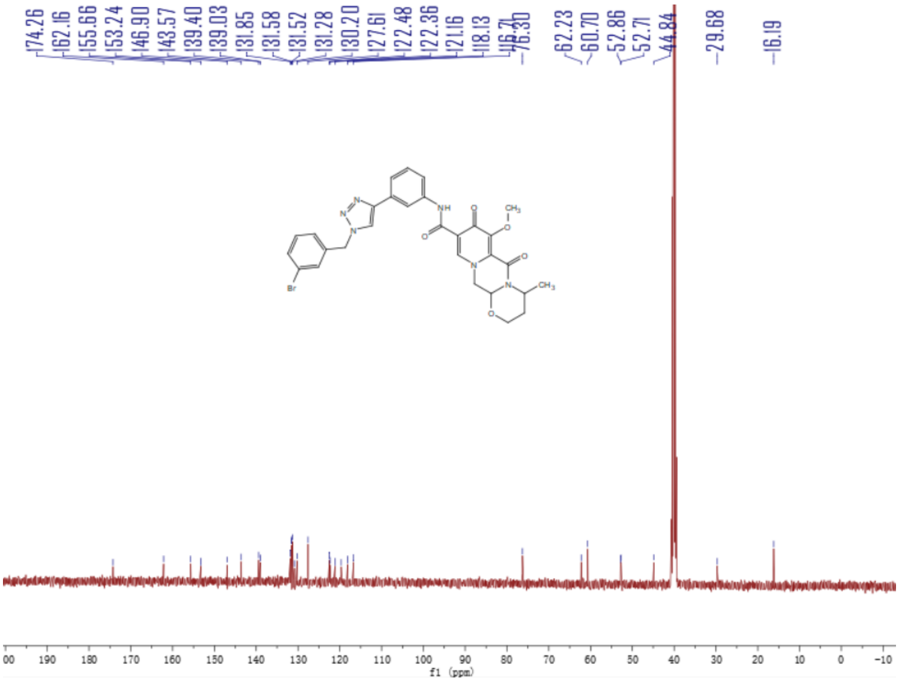

Figure S32. ^{1}H NMR and ^{13}C NMR spectrums of compound 6f

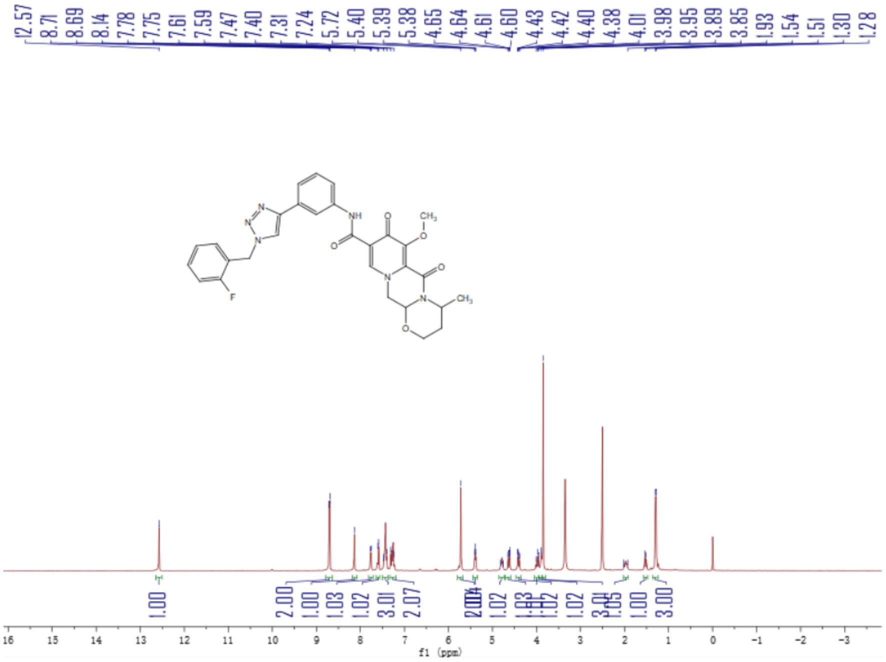

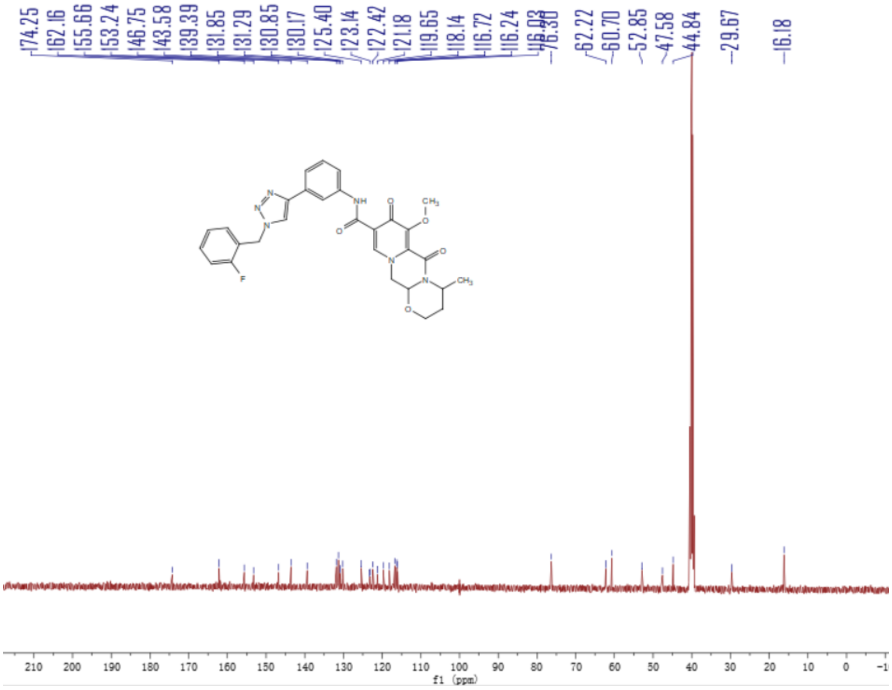

Figure S33. ^{1}H NMR and ^{13}C NMR spectrums of compound 6g

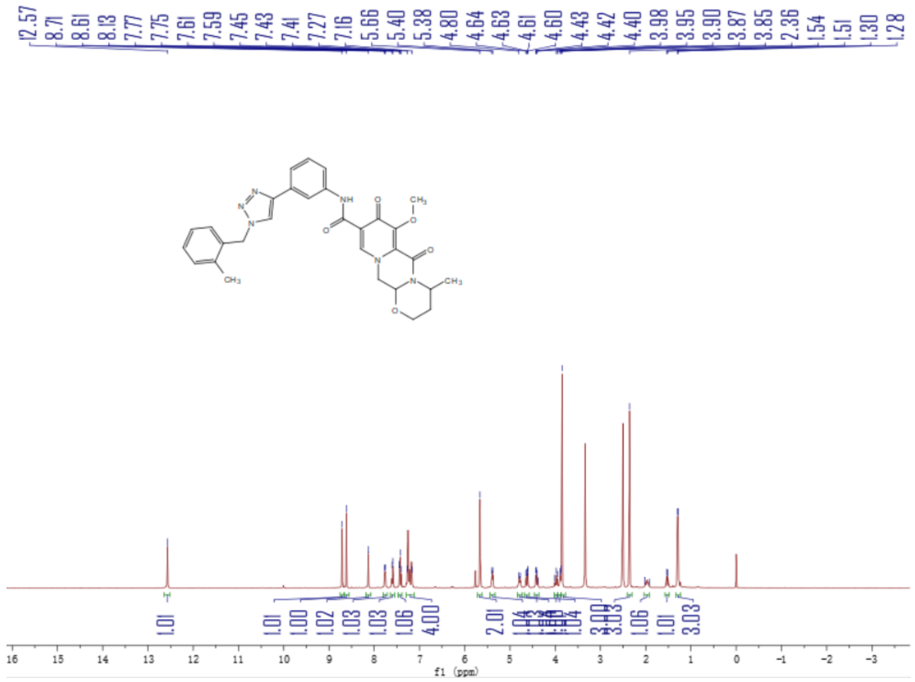

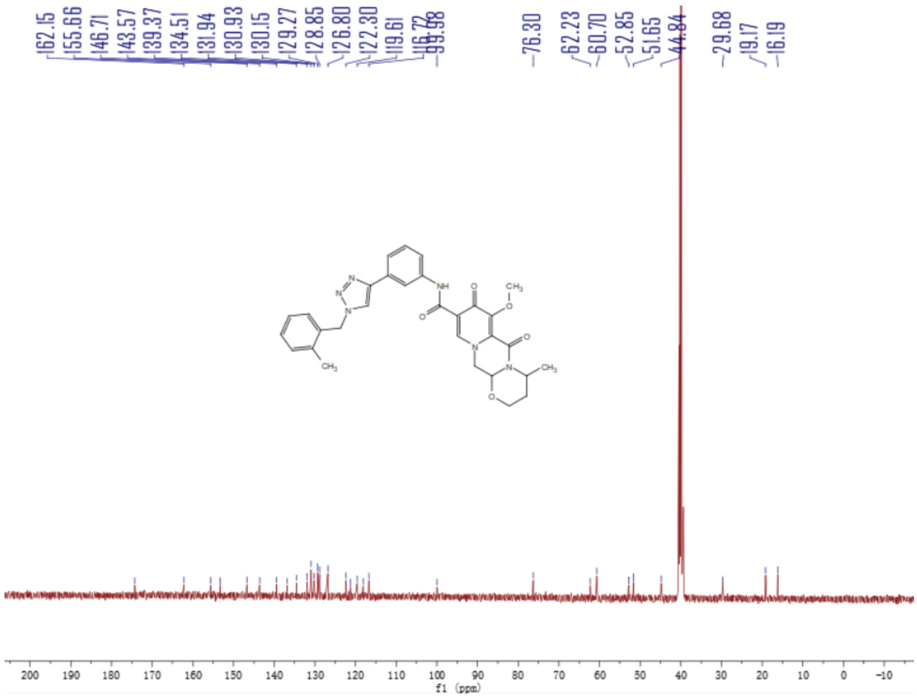

Figure S34. ^{1}H NMR and ^{13}C NMR spectrums of compound 6h

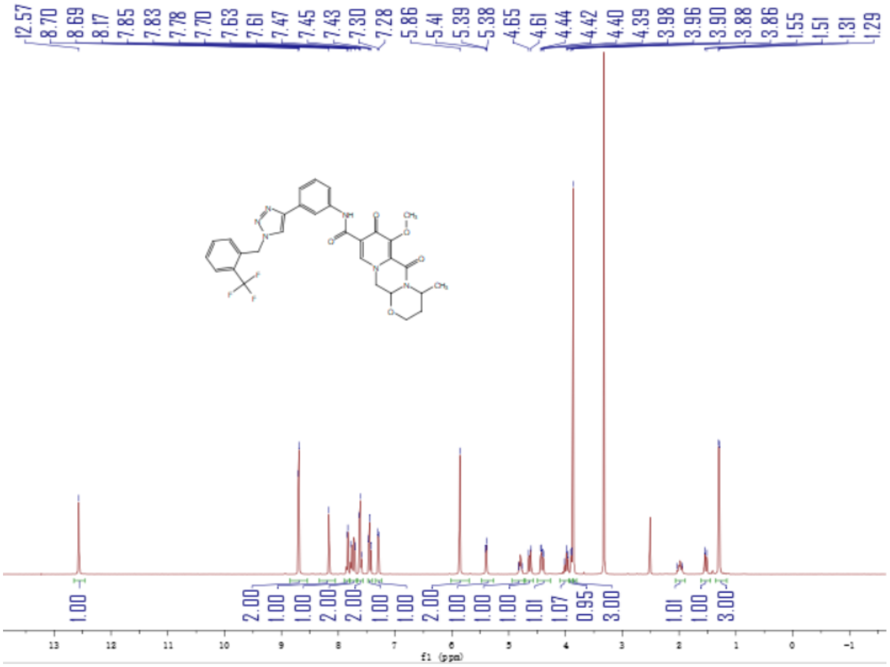

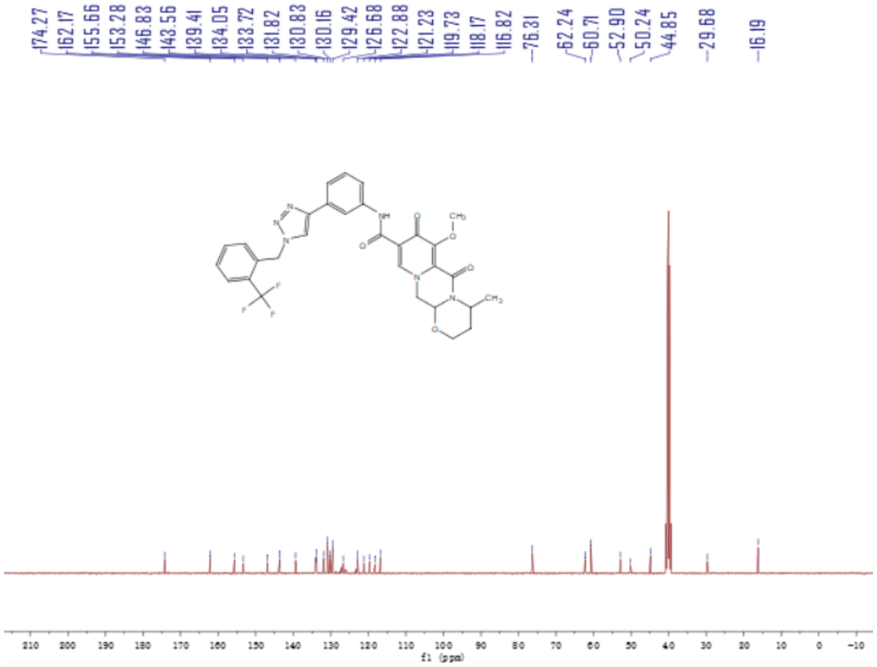

Supplement: Supplementary file 1 [file molecules-29-01779-s001.zip › molecules-2905994-supplementary.pdf]
